# A radial basis function method for noisy global optimisation

**DOI:** 10.1007/s10107-024-02125-9

**Published:** 2024-08-08

**Authors:** Dirk Banholzer, Jörg Fliege, Ralf Werner

**Affiliations:** 1 Department of Mathematical Sciences, University of Southampton, Southampton, SO17 1BJ UK; 2 Institut für Mathematik, Universität Augsburg, 86159 Augsburg, Germany

**Keywords:** Global optimisation, Expensive noisy objective function, Controlled noise, Response surface methods, Radial basis functions, Approximation, 90C26, 90C30

## Abstract

We present a novel response surface method for global optimisation of an expensive and noisy (black-box) objective function, where error bounds on the deviation of the observed noisy function values from their true counterparts are available. The method is based on Gutmann’s well-established RBF method for minimising an expensive and deterministic objective function, which has become popular both from a theoretical and practical perspective. To construct suitable radial basis function approximants to the objective function and to determine new sample points for successive evaluation of the expensive noisy objective, the method uses a regularised least-squares criterion. In particular, new points are defined by means of a target value, analogous to the original RBF method. We provide essential convergence results, and provide a numerical illustration of the method by means of a simple test problem.

## Introduction

In this paper, we are concerned with solving problems of the form

1 $$\begin{aligned} \min _{x \in \mathcal {X}} f(x), \end{aligned}$$

where $\mathcal {X}\subset \mathbb {R}^d$ is a nonempty compact set, and $f:\mathcal {X}\rightarrow \mathbb {R}$ is a continuous potentially nonconvex objective function that is expensive to evaluate. We assume that evaluations of the objective function *f* are perturbed by noise, where the level of noise can be controlled by means of pointwise error bounds. Specifically, given that some noisy function values $\hat{f}(x_i)$ are observed at the sample points $x_i \in \mathcal {X}$, $i \in \mathbb {N}$, we will consider two models for noise:

1. The errors between $\hat{f}(x_i)$ to the true but unknown counterparts $f(x_i)$ can be quantified by
  2 $$\begin{aligned} \big |f(x_i) - \hat{f}(x_i)\big | \le \epsilon _i, \quad i=1,\ldots ,n, \end{aligned}$$
  for some positive values $\epsilon _i$. We denote this case as the case of *fixed noise*.
2. The error bounds can be improved during the course of an iteration, i.e. we consider the $x_1, \ldots , x_n$ as iterates of an optimisation algorithm and presume error bounds of the form
  3 $$\begin{aligned} \big |f(x_i) - \hat{f}^{(n)} (x_i)\big | \le \epsilon _i^{(n)}, \quad i=1,\ldots ,n, \end{aligned}$$
  for some positive errors $\epsilon _i^{(n)}$. We denote this case as the case of *iterative noise*. Iterative noise typically occurs if a function evaluation that computes $\hat{f}^{(n)}(x_i)$ uses, e.g., a Monte Carlo simulation to evaluate some integral occurring in the definition of the function *f*. Given sufficient computational budget, it is then possible to improve a previously computed estimate $\hat{f}^{(k)}(x_i)$ in a later iteration $n > k$ to a hopefully better estimate $\hat{f}^{(n)}(x_i)$ by increasing the sample size of the Monte Carlo simulation. In this paper, we will be concerned with *vanishing iterative noise* in which the $\epsilon _i^{(n)}$ converge to zero for $n \rightarrow \infty$ in some form.

To clarify terminology and avoid any confusion for the purpose of this paper, we define noise to be any inaccuracy in the function evaluation of *f*. In view of problem (1), this is then sometimes also referred to as minimising a noisy objective function $\hat{f}$ on the parameter space $\mathcal {X}$, see, e.g., Kelley [23]. For notational convenience, from now on we always write $\hat{f}^{(n)}$, where in the case of fixed noise we interpret this as $\hat{f}^{(n)} \equiv \hat{f}$. Similarly, we write $\epsilon _i^{(n)}$ for the error bound at the sample point $x_i$, which in the case of fixed noise simplifies to $\epsilon _i^{(n)} \equiv \epsilon _i$.

To effectively tackle the minimisation of a nonconvex and expensive (black-box) objective function, *response surface methods* have been developed. Their basic idea is to approximate the underlying objective function by a sequence of response surface models, i.e. approximants to the function *f*, that guide the selection of new evaluation points to eventually find a global optimum of the original function. To remain easy to handle and cheap to evaluate, the response surface models are usually composed of simple basis functions and fit to the unknown objective function at a limited number of points, either through interpolation or some approximation scheme. Based on the models, new evaluation points are then iteratively determined by some strategy, which ideally balances between selecting points in unexplored regions of the domain to improve the accuracy of the models there, i.e. a global search, and trusting the models in regions with many function evaluations to find a minimum thereof, i.e. a local search. In this way, the models are successively refined to capture the global behaviour of the objective function as best as possible.

Within the class of response surface methods, various methods can be distinguished, see, e.g., Jones [20] or, more recently, Vu et al. [45] for a comparative survey, and Forrester and Keane [7] for a more practical overview. Very generally, one may say that there are three main methodologies according to which traditional response surface methods may be classified.

The underlying idea of *Bayesian methods* is to interpret the objective function *f* as a realisation of a stochastic process $F:\mathcal {X}\times \varOmega \rightarrow \mathbb {R}$, $(x,\omega ) \mapsto F(x,\omega )$, on some probability space $(\varOmega ,\mathcal {F},\mathbb {P})$, such that upon observing $f(x_1),\ldots ,f(x_n)$, the conditional mean function $s_n(x) = \mathbb {E}_\mathbb {P}[F(x) | F(x_1) = f(x_1),\ldots ,F(x_n) = f(x_n)]$ and the variance function $v_n(x) = {{\,\mathrm{\mathbb {V}ar}\,}}_\mathbb {P}(F(x) | F(x_1) = f(x_1),\ldots ,F(x_n) = f(x_n))$ act as a response surface and a measure of the involved error to *f*, respectively. In particular, if the underlying stochastic process is assumed to be Gaussian with mean function $\mathbb {E}_\mathbb {P}[F(x)]$ and covariance function $\mathop {\mathrm {\mathbb {C}ov}}\limits _\mathbb {P}(F(x),F(y))$, $x, y \in \mathcal {X}$, the distributional properties imply that the conditional process is again Gaussian, allowing for suitable expressions in closed form. As for determining new evaluation points, two main strategies can essentially be distinguished in a Bayesian framework. The first strategy, known as P-algorithm, dates back to Kushner [24, 25] and maximises the probability of achieving a certain target function value below the current minimum of the surface in order to find a new point. The second strategy has its origin in Mockus et al. [27] and determines a new point by maximising the expected improvement over the current best function value.

Similar to Bayesian methods, *regression-based methods*, which are commonly referred to as Kriging [26], also assume *f* to be a realisation of a stochastic process $\{F(x)\}_{x\in \mathcal {X}}$ but use a linear regression to fit a response surface model. Specifically, given the observations $f(x_1),\ldots ,f(x_n)$, these methods derive a response surface $s_n$ as the best linear unbiased predictor and the corresponding error $v_n$ as the mean squared error, see, e.g., Sacks et al. [35] for more details, thus leading to the same methods as in the Bayesian methodology for the special case of a Gaussian process (see, e.g., Fowkes [8] for the equivalence), but otherwise different ones. The most popular method embedded within a regression-based methodology is the Efficient Global Optimisation (EGO) algorithm by Jones et al. [22], which specifies the covariance structure of the stochastic process between sampled points by a Gaussian correlation function and finds the next evaluation point by using the expected improvement criterion, as suggested for Bayesian methods. Schonlau [40] observes that the latter criterion, being independent of any parameter, may result in a search that overly emphasises local search and suggests using a generalised expected improvement, which introduces an additional parameter that controls the balance between global versus local search. Another related modification that allows to exogenously control local and global search by an additional parameter is the weighted expected improvement, due to Sóbester et al. [42]. The EGO approach to construct suitable response surfaces has also been used by Villemonteix et al. [44] in their Informational Approach to Global Optimisation (IAGO). However, instead of expected improvement, they use the conditional entropy of a minimiser as a criterion to iteratively determine new evaluation points. Further work allow for constraints [13], multiobjective problems [6], and parallelisation [46].

For methods that do not model the objective function by means of stochastic processes, a *general response surface technique* for finding a new evaluation point is proposed by Jones [19]. It assumes the existence of a linear space of functions $\mathcal {A}$, which is left unspecified but admits a measure of ‘bumpiness’ $\sigma (s)$ for its elements $s \in \mathcal {A}$. In any given iteration, once a response surface model has been fitted to a set of function values $f(x_1),\ldots ,f(x_n)$ through interpolation, a target value $f^*$ is then chosen which may be considered as a rough estimate of the global minimum of *f*. By this choice, a new evaluation point $x_{n+1}$ is then determined as the value of $y \notin \{x_1,\ldots ,x_n\}$ such that the augmented response surface $s_y \in \mathcal {A}$ minimises the bumpiness $\sigma (s)$ on $\mathcal {A}$, subject to the interpolation conditions

$$\begin{aligned} s_y(x_i)&= f(x_i), \qquad i=1,\ldots ,n, \\ s_y(y)&= f^*, \end{aligned}$$

and provided that, for any $y \in \mathcal {X}\backslash \{x_1,\ldots ,x_n\}$, the interpolant $s_y \in \mathcal {A}$ is uniquely defined. The most prominent response surface method that is based on Jones’ general technique is suggested by Gutmann [10, 12] in form of the radial basis function (RBF) method. As the name suggests, the method relies on the use of radial basis functions, which not only ensures the uniqueness of interpolants under relatively mild conditions on the location of the sample points, but also provides in a natural way a measure of ‘bumpiness’ in form of a semi-norm. The strategy for determining new evaluation points is based on a mathematically sound mechanism, which facilitates establishing convergence of the method and its close relation to the P-algorithm. On the practical side, the method has proven to be a powerful tool and performs well on well-behaved expensive optimisation problems, see, e.g., Björkman and Holmström [2], while the noted slow convergence of the method to a global minimum of more complex objective functions has been addressed by Regis and Shoemaker [32], Holmström [14], and Cassioli and Schoen [4], most notably. In addition, Costa and Nannicini [5] propose a technique to speed up the practical convergence of Gutmann’s RBF method in case noisy and less expensive function values are additionally available.

Finally, other works using radial basis functions to construct response surfaces in a deterministic setup include, for instance, the investigation of the multiobjective case [1, 29, 47] and the case of black-box constraints [28]. Also, there are approaches which seemingly do not rely on any of the described underlying methodologies and are thus designed to work with any kind of response surface model, see, e.g, Regis and Shoemaker [30, 31, 33, 34] and Ji et al. [18].

Despite its importance in applications, the global optimisation of expensive objective functions in the presences of noise has attracted considerably less attention than the equivalent optimisation without noise. Due to the underlying probabilistic framework that is provided by Bayesian and regression-based methods, the fundamental derivations of response surfaces and corresponding error measures, i.e. of the conditional mean and variance functions as well as of the unbiased predictors and mean squared errors, respectively, can be extended straightforwardly to noisy observations, by adding randomly distributed error terms to the modelling stochastic process, see, e.g., Schonlau [40]. Yet, the determination of new evaluation points poses a substantial difficulty, which has been dealt with differently by a few authors. In their Sequential Kriging Optimisation (SKO) method, Huang et al. [15] extend the EGO algorithm to noisy objective function values, assuming that the involved random errors are i.i.d. normally distributed with constant variance. Correspondingly, the method relies on the same Gaussian covariance structure of the EGO algorithm plus a variance term, and to select new evaluation points, an augmented expected improvement criterion is derived that calculates a scaled expected improvement over the response surface value of the so-called effective best solution, instead of the current minimum function value. A further extension to the expected improvement criterion that may be used in a Bayesian/regression-based setup for noisy objective function values is suggested by Gramacy and Lee [9], also known as the integrated expected conditional improvement. Their main idea is to consider the expected improvement at a reference point, given that the objective function has been sampled at a candidate point, i.e. the expected conditional improvement, and find the next point as the maximiser of this criterion, integrated by a suitable density function over all reference points. Finally, in Villemonteix et al. [44], the authors also show that their IAOG method for exact function values can be extended to handle noisy observations. Specifically, they assume that the errors in the observed function values are i.i.d. normally distributed with known mean and variance, and estimate the conditional entropy criterion by simulating on the noisy observed function values, instead of the true ones.

Relating to Jones’ general technique in the presence of noise, Žilinskas [51] addresses the similarity between the P-algorithm and Gutmann’s RBF method and shows that these techniques can be extended to noisy function values if appropriate modifications are made in both algorithms. In particular, he suggests to construct radial basis function approximants for the latter by minimising the semi-norm such that the residual sum of squares of an approximant to the noisy observations is proportional to the variance of the involved additive noise, which is assumed to be constant and known. New evaluation points may then be determined similar to Jones’ technique by means of target values, i.e. by minimising the semi-norm of an augmented surface such that it interpolates a chosen target value and such that the residual sum of squares of the augmented surface to the noisy observations is proportional to the known variance. However, even though Žilinskas establishes the theoretical similarity between the P-algorithm and the RBF method in a noisy setup, no explicit algorithm making use of this result is proposed. Radial basis functions are also used in the algorithm by Jakobsson et al. [17], called qualSolve, for the global optimisation of expensive black-box functions subject to noise. Here, the authors construct response surfaces by minimising the convex sum of the squared semi-norm of a radial basis function approximant and the squared difference between its values at the sample points and the noisy observations, where an additionally introduced parameter to balance between both measures is estimated by cross validation. To select new evaluation points a quality function is maximised, which is calculated at each point by the minimum distance to previously evaluated points and weighted by the response surface value at that point. In particular, the weights are adjusted periodically in order to alternate between local and global search of the method.

Finally, Shen and Shoemaker [41] build radial basis approximants to functions with homoscedastic noise by minimising the weighted squared semi-norm and the sum of squared differences between response surface values and observations. A new evaluation point is then selected in each iteration from a set of randomly generated candidate points by a weighted score that balances between exploration and exploitation of the parameter space.

Given above contributions, we present in this paper a novel RBF method for noisy objective functions in which the level of noise can be controlled by means of pointwise error bounds. The method is essentially based on Gutmann’s original RBF method for deterministic objective functions and uses some of the ideas from Žilinskas [51] to extending Gutmann’s method to a noisy setup. In establishing the method, we address the construction of appropriate response surfaces and the determination of new evaluation points once a surface has been constructed, as these are the main components that require specification in order to deal with noisy function values. In particular, since radial basis function interpolation is no longer feasible in the present situation, we first consider common approaches for the approximation of a noisy function by means of radial basis functions and briefly discuss their suitability for integration into a response surface method. As regularised least-squares approximants explicitly seek to balance between the bumpiness of the surface and the closeness to the data, where the additional regularisation parameter may be set in accordance with the available error bounds, they turn out to be particularly suited for our purposes. Moreover, the least-squares criterion allows for a convenient adaption of Jones’ technique to determine new evaluation points through target values, by analogy with Gutmann’s original algorithm. In particular, this functionality also allows to establish convergence of the method, where we show that the convergence properties of Gutmann’s deterministic method are kept when the exact function values are replaced by corresponding noisy values. As we will see, convergence can be achieved by updating the regularisation parameter in a particular way, depending on the model of noise:

1. In the case of fixed noise, it is sufficient to ensure that the sequence of regularisation parameters converges to zero quickly enough. As such, it suffices to choose these parameters according to some exogenous sequence, at least for theoretical purposes. This result follows from Theorem 2 (p. 22) together with Theorem 5 (p. 26).
2. In the case of iterative noise, if the noise vanishes fast enough, it is possible to choose the regularisation parameter in each step in such a way that the bumpiness of the approximant is as small as possible, thereby greatly simplifying the inner optimisation step in which an augmented function based on this approximant is minimised. This follows from Theorems 2, 3 (p. 22), and 5.

The remainder of this paper is organised as follows. In Sect. 2, we review Gutmann’s original RBF method to minimise a deterministic nonconvex objective function that is expensive to evaluate. In Sect. 3, we briefly outline common approaches for radial basis function approximation and discuss their suitability for integration into a response surface method. Based on regularised least-squares approximants, we then present in Sect. 4 a RBF method for minimising a noisy nonconvex and expensive objective function, given that error bounds on the observed function values are available. In Sect. 5, we establish the convergence of the method. In Sect. 6, we provide a numerical illustration of the proposed RBF method by means of a simple test problem, while Sect. 7 contains our conclusions.

For ease of reference, we provide in Table 1 the most relevant variables and further notations, together with their meaning.

**Table 1:** List of main notations used throughout this paper

Notation	Meaning
*f*	Continuous, potentially nonconvex, objective function from $\mathbb {R}^d$ into $\mathbb {R}$
$\mathcal {X}$	Compact parameter space in $\mathbb {R}^d$
$\hat{f}^{(n)}(x_i)$	Noisy function value of *f* at point $x_i$ in the *n*-th iteration of the RBF method for noisy objectives functions
$\epsilon _i^{(n)}$	Error bound at point $x_i$ in the *n*-th iteration of the RBF method for noisy objective functions
$\phi$	Radial basis function
$\mathcal {P}_m^d$	Space of polynomials of total degree at most $m-1$ in $\mathbb {R}^d$
${\widetilde{m}}$	Dimension of $\mathcal {P}_m^d$, i.e. $${\widetilde{m}}= \left( {\begin{array}{c}m + d\\ m\end{array}}\right) $$
$\mathcal {F}_{\phi ,m}(\mathcal {X})$	Space of linear combinations of radial basis functions $\phi (\Vert {\hspace{1.111pt}\cdot \hspace{1.111pt}}- x\Vert _2)$, $x \in \mathcal {X}$
$\mathcal {A}_{\phi , m}(\mathcal {X})$	Direct sum of $\mathcal {F}_{\phi ,m}(\mathcal {X})$ and $\mathcal {P}_m^d$
$\Vert {\hspace{1.111pt}\cdot \hspace{1.111pt}}\Vert _\phi$	Semi-norm on $\mathcal {A}_{\phi , m}(\mathcal {X})$, induced by the semi-inner product $\langle {\hspace{1.111pt}\cdot \hspace{1.111pt}}{,}{\hspace{1.111pt}\cdot \hspace{1.111pt}}\rangle _{\phi }$
$s_n$	Interpolant from $\mathcal {A}_{\phi , m}(\mathcal {X})$ to the data $(x_1, f(x_1))$, ..., $(x_n, f(x_n))$
$l_n(y, \cdot )$	Interpolant from $\mathcal {A}_{\phi , m}(\mathcal {X})$ to the data $(x_1, 0)$, ..., $(x_n, 0)$ and (*y*, 1)
$f^{*}_n$	Target value in the *n*-th iteration of the RBF method for deterministic and noisy objective functions
$\mu _n$, $g_n$	Functions on $\mathcal {X}\backslash \{x_1,\ldots , x_n\}$ to be minimised for obtaining $x_{n+1}$
$v_n$, $h_n$	Utility functions on $\mathcal {X}\backslash \{x_1,\ldots , x_n\}$, identical to $1/\mu _n$ and $1/g_n$, respectively
$\varDelta _n(y)$	Function assigning the minimum Euclidean distance of $y \in \mathcal {X}$ to the set $\{x_1,\ldots ,x_n\}$
$s^{\gamma _n}_n$	Regularised least-squares approximant with parameter $\gamma _n$ from $\mathcal {A}_{\phi , m}(\mathcal {X})$ to the data $(x_1, \hat{f}^{(n)}(x_1))$, ..., $(x_n, \hat{f}^{(n)}(x_n))$
$\gamma _n$	Regularisation parameter, weight for $\Vert s\Vert _\phi ^2$ in the construction of the regularised least-squares approximant $s_n^{\gamma _n}$
$w_i$	Weight for $(s(x_i) - \hat{f}^{(n)}(x_i) )^2$ in the construction of the regularised least-squares approximant $s_n^{\gamma _n}$
$l_n^{\gamma _n}(y, \cdot )$	Regularised least-squares approximant with parameter $\gamma _n$ from $\mathcal {A}_{\phi , m}(\mathcal {X})$ to the data $(x_1, 0)$, ..., $(x_n, 0)$, subject to interpolating (*y*, 1)
$\mu _n^{\gamma _n}$, $g_n^{\gamma _n}$	Functions with parameter $\gamma _n$ on $\mathcal {X}\backslash \{x_1, \ldots , x_n\}$ (can be continuously extended at $x_1,\ldots ,x_n$) to be minimised for obtaining $x_{n+1}$
$v_n^{\gamma _n}$, $h_n^{\gamma _n}$	Utility functions with parameter $\gamma _n$ on $\mathcal {X}\backslash \{x_1, \ldots , x_n\}$ (can be continuously extended at $x_1,\ldots ,x_n$), identical to $1/\mu _n^{\gamma _n}$ and $1/g_n^{\gamma _n}$, respectively
$\widetilde{w}_n(y)$	Function assigning the weight $w_i$ of the sample point $x_i$, $i \in \{1,\ldots , n\}$, that is closest to $y \in \mathcal {X}$

## Gutmann’s RBF method

Let us briefly describe Gutmann’s original RBF method [10, 12] for deterministic objective functions, as this will provide us with the necessary tools to generalise this method to the noisy case considered in this paper. The method relies on the general technique by Jones [19], but specifically employs radial functions to construct response surface interpolants of the generic form

4 $$\begin{aligned} s(x) = \sum _{i=1}^n \lambda _i \phi (\Vert x-x_i\Vert _2) + p(x), \quad x \in \mathbb {R}^d, \end{aligned}$$

where $\phi :[0,\infty ) \rightarrow \mathbb {R}$ is a fixed radial function, $\{\lambda _i\}_{i=1}^n$ are real coefficients, $\{x_i\}_{i=1}^n \subset \mathbb {R}^d$ are distinct centre points, and $p \in \mathcal {P}_m^d$ is a polynomial from the linear space of all real-valued polynomials of total degree at most $m-1$ in *d* variables, with $\mathcal {P}_0^d = \{0\}$. On the linear space of all functions of the form (4) on $\mathcal {X}$, formally defined by

5 $$\begin{aligned} \mathcal {A}_{\phi , m} (\mathcal {X}):= \mathcal {F}_{\phi ,m}(\mathcal {X}) + \mathcal {P}_m^d \end{aligned}$$

with

$$\begin{aligned} \begin{aligned} \mathcal {F}_{\phi ,m}(\mathcal {X})&:= \Bigg \{\sum _{i=1}^n \lambda _i \phi (\Vert {\hspace{1.111pt}\cdot \hspace{1.111pt}}- x_i\Vert _2): n \in \mathbb {N}, \lambda \in \mathbb {R}^n, \{x_i\}_{i=1}^n \subset \mathcal {X}, \\&\quad \sum _{i=1}^n \lambda _i p(x_i) = 0,\, p \in \mathcal {P}_m^d \Bigg \}, \end{aligned} \end{aligned}$$

a measure of ‘bumpiness’ is then given in a natural way by the semi-norm $\Vert {\hspace{1.111pt}\cdot \hspace{1.111pt}}\Vert _\phi := \langle {\hspace{1.111pt}\cdot \hspace{1.111pt}}{,}{\hspace{1.111pt}\cdot \hspace{1.111pt}}\rangle _\phi ^{1/2}$, induced by the semi-inner product

6 $$\begin{aligned} \langle s,u \rangle _\phi := \sum _{i=1}^{n(s)} \lambda _i^s u(x_i^s), \end{aligned}$$

for any two elements $s, u\in \mathcal {A}_{\phi , m} (\mathcal {X})$ with

$$\begin{aligned} s(x) = \sum _{i=1}^{n(s)} \lambda _i^s \phi (\Vert x - x_i^s\Vert _2) + p^s(x) \quad \text {and} \quad u(x) = \sum _{i=1}^{n(u)} \lambda _i^u \phi (\Vert x - x_i^u\Vert _2) + p^u(x). \end{aligned}$$

The classical choices of radial basis functions $\phi$, along with their minimal order ${m_\phi }$ guaranteeing conditional positive definiteness are given in Table 2, cf. [12], listing (3.2). In what follows, we will also need the notion of *conditionally positive definite functions*. Recall that a continuous radial function $\phi$ is conditionally positive definite of order *m* if $\sum _{i=1}^n \sum _{j=1}^n \lambda _i \lambda _j \phi (\Vert x_i - x_j\Vert _2) > 0$ for any pairwise distinct points $x_1,\ldots ,x_n$, $n \in \mathbb {N}$, and any $\lambda \in \mathbb {R}^n \backslash \{0\}$ satisfying $\sum _{i=1}^n \lambda _i p(x_i) = 0$, $p \in \mathcal {P}_m^d$. Since a conditionally positive definite function of order $m_1$ is also conditionally positive definite of order $m_2 \ge m_1$, particular interest is given to the smallest possible order ${m_\phi }\in \mathbb {N}_0$ such that $\phi$ is conditionally positive definite.

**Table 2:** Common choices of radial basis functions, their shape parameter $\zeta >0$, smoothing parameter $\nu$, and the minimal order ${m_\phi }$

Radial basis function	$\phi (r)$	Specification	Minimal order ${m_\phi }$
Surface splines	$r^\nu$	$\nu \in \mathbb {N}$, $\nu$ odd	$\lfloor \nu /2\rfloor + 1$
	$r^\nu \log {r}$	$\nu \in \mathbb {N}$, $\nu$ even	
Multiquadrics	$(r^2 + \zeta ^2)^\nu$	$\nu > 0$, $\nu \not \in \mathbb {N}$	$\lfloor \nu \rfloor + 1$
Inverse multiquadrics	$(r^2 + \zeta ^2)^\nu$	$\nu < 0$	0
Gaussians	$e^{-\zeta r^2}$		0

In the following, let $\phi$ be a conditionally positive definite radial basis function of order $m$, and let $\{p_j\}_{j=1}^{\widetilde{m}}$ be a basis of the space of polynomials $\mathcal {P}_m^d$ of degree at most $m-1$ with dimension ${\widetilde{m}}$.

### Construction of response surface

Suppose we are in iteration *n* of the algorithm and can interpolate the data $(x_1, f(x_1)),\ldots ,(x_n, f(x_n))$. To construct an interpolant $s_n \in \mathcal {A}_{\phi , m} (\mathcal {X})$ of the form (4), with $\mathcal {A}_{\phi , m} (\mathcal {X})$ defined as in (5), the corresponding coefficients are determined by solving

7 $$\begin{aligned} \begin{aligned} \min _{s \in \mathcal {A}_{\phi , m} (\mathcal {X})} \; \Vert s\Vert _\phi \quad \text {s.t.} \quad s(x_i) = f(x_i), \quad i=1,\ldots ,n, \end{aligned} \end{aligned}$$

which reduces to solving the linear system (see Schaback [36])

8 $$\begin{aligned} \begin{pmatrix} \varPhi &{} P \\ P^\top &{} 0 \end{pmatrix} \begin{pmatrix} \lambda \\ c \end{pmatrix} = \begin{pmatrix} F \\ 0 \end{pmatrix}, \end{aligned}$$

where $\varPhi \in \mathbb {R}^{n \times n}$ and $P \in \mathbb {R}^{n \times {\widetilde{m}}}$ denote the interpolation and polynomial basis matrix with entries $\varPhi _{ij} = \phi (\Vert x_i-x_j\Vert _2)$, $i, j = 1,\ldots ,n$, and $P_{ij} = p_j(x_i)$, $i=1,\ldots ,n$, $j=1,\ldots ,{\widetilde{m}}$, respectively, $\lambda \in \mathbb {R}^n$ and $c \in \mathbb {R}^{\widetilde{m}}$ are the coefficient vectors, and $F = (f(x_1),\ldots ,f(x_n))^\top$ stands for the vector of observed function values.

The unique solvability of the linear system (8) follows under the relatively mild condition that the sample points $x_1,\ldots ,x_n$ form a $\mathcal {P}_m^d$-unisolvent set, i.e. if $p \in \mathcal {P}_m^d$ and $p(x_i) = 0$, $i = 1,\ldots ,n$, then $p \equiv 0$, see, e.g., Wendland [49] for details. Moreover, it is easy to verify that the linear system will remain uniquely solvable upon the successive addition of new data points, provided that they are distinct from previous ones.

#### Determination of next evaluation point

Upon the construction of $s_n$, the next evaluation point $x_{n+1}$ is determined according to Jones’ general technique. More precisely, for a given target value $f_n^*$ that will be specified further below, the point $x_{n+1}$ is given as the point $y \in \mathcal {X}\backslash \{x_1,\ldots ,x_n\}$ such that there is an *augmented surface* $s_y \in \mathcal {A}_{\phi , m} (\mathcal {X})$ that solves

9 $$\begin{aligned} \begin{aligned} \min _{\begin{array}{c} {y \in \mathcal {X}}\\ {s \in \mathcal {A}_{\phi , m} (\mathcal {X})} \end{array}} \; \Vert s\Vert _\phi \quad \text {s.t.} \quad s(x_i)&= f(x_i), \quad i=1,\ldots ,n, \\ s(y)&= f_n^*. \end{aligned} \end{aligned}$$

To simplify problem (9), the optimal interpolant $s_y \in \mathcal {A}_{\phi , m} (\mathcal {X})$, $y \in \mathcal {X}\backslash \{x_1,\ldots ,x_n\}$, satisfying the interpolation conditions in (9) can be rewritten as

10 $$\begin{aligned} s_y(x) = s_n(x) + \big [f_n^*- s_n(y) \big ] l_n(y,x), \quad x \in \mathbb {R}^d, \end{aligned}$$

where $l_n(y,{\hspace{1.111pt}\cdot \hspace{1.111pt}}) \in \mathcal {A}_{\phi , m} (\mathcal {X})$ is the optimal interpolant to

11 $$\begin{aligned} \begin{aligned} l_n(y,x_i)&= 0, \qquad i=1,\ldots ,n, \\ l_n(y,y)&= 1. \end{aligned} \end{aligned}$$

In particular, the function $l_n(y,{\hspace{1.111pt}\cdot \hspace{1.111pt}})$ can be expressed as

$$\begin{aligned} l_n(y,x) = \sum _{i=1}^n \alpha _i(y) \phi (\Vert x-x_i\Vert _2) + \beta (y) \phi (\Vert x-y\Vert _2) + \sum _{j=1}^{\widetilde{m}}b_j(y) p_j(x), \quad x \in \mathbb {R}^d, \end{aligned}$$

whose coefficients $\alpha (y) = (\alpha _1(y),\ldots ,\alpha _n(y))^\top \in \mathbb {R}^n$, $\beta (y) \in \mathbb {R}$, and $b(y) = (b_1(y),\ldots ,b_{\widetilde{m}}(y))^\top \in \mathbb {R}^{\widetilde{m}}$ solve the linear system

12 $$\begin{aligned} \begin{pmatrix} \varPhi &{} u_n(y) &{} P \\ u_n(y)^\top &{} \phi (0) &{} \pi (y)^\top \\ P^\top &{} \pi (y) &{} 0 \end{pmatrix} \begin{pmatrix} \alpha (y) \\ \beta (y) \\ b(y) \end{pmatrix} = \begin{pmatrix} 0 \\ 1 \\ 0 \end{pmatrix}, \end{aligned}$$

for the matrices $\varPhi \in \mathbb {R}^{n \times n}$ and $P \in \mathbb {R}^{n \times {\widetilde{m}}}$, and the vectors $u_n(y):= (\phi (\Vert x_1-y\Vert _2),\ldots ,\phi (\Vert x_n-y\Vert _2))^\top \in \mathbb {R}^n$ and $\pi (y):= (p_1(y),\ldots ,p_{\widetilde{m}}(y))^\top \in \mathbb {R}^{\widetilde{m}}$. By means of representation (10), the squared semi-norm of $s_y$ can then be simplified to

13 $$\begin{aligned} \Vert s_y\Vert _\phi ^2&= \Vert s_n\Vert _\phi ^2 + 2 \big [ f_n^*- s_n(y)\big ] \langle s_n, l_n(y,{\hspace{1.111pt}\cdot \hspace{1.111pt}}) \rangle _\phi + \big [ f_n^*- s_n(y)\big ]^2 \Vert l_n(y,{\hspace{1.111pt}\cdot \hspace{1.111pt}})\Vert _\phi ^2 \nonumber \\&= \Vert s_n\Vert _\phi ^2 + \beta (y) \big [ f_n^*- s_n(y)\big ]^2, \end{aligned}$$

using the definition of the semi-inner product (6) and the interpolation conditions (11). Since $\Vert s_n\Vert _\phi$ is independent of *y*, Eq. (13) shows that the required minimisation of $\Vert s_y\Vert _\phi$ with respect to *y* boils down to minimising the nonnegative function

14 $$\begin{aligned} g_n(y) := \mu _n(y) \big [ f_n^*- s_n(y) \big ]^2, \qquad y \in \mathcal {X}\backslash \{x_1,\ldots ,x_n\}, \end{aligned}$$

where the function $\mu _n:\mathcal {X}\backslash \{x_1,\ldots ,x_n\} \rightarrow \mathbb {R}$ is given by

15 $$\begin{aligned} \mu _n(y) := \Vert l_n(y,{\hspace{1.111pt}\cdot \hspace{1.111pt}})\Vert _\phi ^2 = \beta (y). \end{aligned}$$

Note that the function $\mu _n$ is well-defined and allows for the properties described in the following two remarks.

##### Remark 1

Definition (15) provides that $\mu _n(y) > 0$ for $y \in \mathcal {X}\backslash \{x_1,\ldots ,x_n\}$: Assuming there is an $y_0 \in \mathcal {X}\backslash \{x_1,\ldots ,x_n\}$ with $\mu _n(y_0) = 0$, definition (15) and the $\mathcal {P}_m^d$-unisolvency of $\{x_1,\ldots ,x_n\}$ yield $l_n(y_0,{\hspace{1.111pt}\cdot \hspace{1.111pt}}) \equiv 0$. This, however, is in contradiction to the interpolation constraint $l_n(y_0,y_0) = 1$.

##### Remark 2

By applying Cramer’s rule to the linear system (12), the function $\mu _n$ can be computed as

$$\begin{aligned} \mu _n(y) = \frac{\det {A}_n}{\det {A_n(y)}}, \qquad y \in \mathcal {X}\backslash \{x_1,\ldots ,x_n\}, \end{aligned}$$

where $A_n$ and $A_n(y)$ are given by the nonsingular interpolation matrices on the left-hand sides of Eqs. (8) and (12), respectively. Hence, since $\det A_n$ is a nonzero constant and $\lim _{y \rightarrow x_i} \det A_n(y) = 0$ for any $i \in \{1,\ldots ,n\}$, it follows that

$$\begin{aligned} \lim _{y \rightarrow x_i} \mu _n(y) = \infty , \qquad i=1,\ldots ,n. \end{aligned}$$

Also note that the function $\mu _n$ (and thus $g_n$) can be equivalently expressed, cf. [12], Proposition 4.12, which allows for a more intuitive interpretation as well as a more efficient computation.

##### Remark 3

The function defined by

$$\begin{aligned} v_n(y) := \Bigg [ \phi (0) - \begin{pmatrix} u_n(y) \\ \pi (y) \end{pmatrix}^\top \begin{pmatrix} \varPhi &{} P \\ P^\top &{} 0 \end{pmatrix}^{-1} \begin{pmatrix} u_n(y) \\ \pi (y) \end{pmatrix} \Bigg ], \qquad y \in \mathbb {R}^d, \end{aligned}$$

is identical to $1/\mu _n(y)$ on $y \in \mathbb {R}^d\backslash \{x_1,\ldots ,x_n\}$. In particular, since $v_n$ is zero at the sample points $x_1,\ldots ,x_n$ and positive and finite elsewhere, it provides a measure of the uncertainty of the model $s_n$ to *f*. Moreover, based on $v_n$, the function defined by

$$\begin{aligned} h_n(y) := \frac{v_n(y)}{\big [s_n(y) - f_n^*\big ]^2}, \qquad y \in \mathbb {R}^d, \end{aligned}$$

is identical to $1/g_n(y)$ on $y \in \mathbb {R}^d\backslash \{x_1,\ldots ,x_n\}$.

#### Choice of target value

The choice of $f_n^*$ crucially influences the location of the new point $x_{n+1}$. To guarantee that $x_{n+1}$ as a global minimiser of $g_n$ on $\mathcal {X}\backslash \{x_1,\ldots ,x_n\}$ exists and does not coincide with previous sample points, it must hold that

16 $$\begin{aligned} f_n^*\in \bigg [-\infty ,\min _{y \in \mathcal {X}} s_n(y) \bigg ], \end{aligned}$$

where the case $f_n^*= \min _{y \in \mathcal {X}} s_n(y)$ is only admissible if $f_n^*< s_n(x_i)$, $i=1,\ldots ,n$.

Specifically, for low target values satisfying (16), the method essentially performs a global search in which the new point $x_{n+1}$ is sampled away from already evaluated points. A high target value close or equal to $\min _{y \in \mathcal {X}} s_n(y)$ is supposed to sample $x_{n+1}$ either in the vicinity of a global minimiser of $s_n$, if $f_n^*< \min _{y \in \mathcal {X}} s_n(y)$, or as a global minimiser of $s_n$, if $f_n^*= \min _{y \in \mathcal {X}} s_n(y)$, cf. Regis and Shoemaker [32]. In particular, for $f_n \rightarrow -\infty$, one can observe by the definition of $g_n$ and the boundedness of $s_n$ on $\mathcal {X}$ that $\mu _n(x_{n+1}) \le \mu _n(y)$, $y \in \mathcal {X}\backslash \{x_1,\ldots ,x_n\}$. Hence, choosing $f_n^*= -\infty$ reduces the minimisation of $g_n$ on $\mathcal {X}\backslash \{x_1,\ldots ,x_n\}$ to the minimisation of $\mu _n$, which samples $x_{n+1}$ as far away as possible from the points $x_1,\ldots ,x_n$.

#### Summary of Gutmann’s RBF method

Altogether, Gutmann’s RBF method for minimising a deterministic and continuous function $f:\mathcal {X}\rightarrow \mathbb {R}$ on a compact set $\mathcal {X}$ can be summarised as follows.

##### Algorithm 1

(Gutmann’s RBF Method).

0. **Initial step:**
  Choose a conditionally positive definite radial basis function $\phi$ of order $m$.
  Generate a $\mathcal {P}_m^d$-unisolvent set of points $\{x_1,\ldots ,x_{n_0}\} \subset \mathcal {X}$.
  Evaluate *f* at the points $x_1,\ldots ,x_{n_0}$, and set $n=n_0$.
1. **Iteration step:**
  **while** $$n \le n^{\max }$$ **do**
    Construct the interpolant $s_n \in \mathcal {A}_{\phi , m} (\mathcal {X})$ solving
      $$\begin{aligned} \min _{s \in \mathcal {A}_{\phi , m} (\mathcal {X})} \Vert s\Vert _\phi \qquad \text {s.t.} \quad s(x_i)=f(x_i), \quad i=1,\ldots ,n. \end{aligned}$$
    Choose an admissible target value $f_n^*\in \big [-\infty , \min _{y \in \mathcal {X}} s_n(y) \big ]$.
    Determine $x_{n+1}$, which is the value of *y* that solves
      $$\begin{aligned} \min _{y \in \mathcal {X}\backslash \{x_1,\ldots ,x_n\}} \mu _n(y)\big [f_n^*- s_n(y)\big ]^2. \end{aligned}$$
    Evaluate *f* at $x_{n+1}$, and set $n=n+1$.
  **end while**

## Approximation with radial basis functions

To recover an unknown function $f:\mathcal {X}\rightarrow \mathbb {R}$ on some set $\mathcal {X}\subset \mathbb {R}^d$ from a number of observed function values $f(x_1),\ldots ,f(x_n)$ with $x_1,\ldots ,x_n \in \mathcal {X}$, an interpolation technique is typically adopted if the respective function values are known to be exact. However, if the observations are contaminated by noise, i.e. we observe $\hat{f}^{(n)} (x_1), \ldots , \hat{f}^{(n)}(x_n)$ in step *n* of our algorithm, then other approximation techniques are recommended. In particular, if an interpolation was used for noisy function observations, too much weight would be given to the involved noise, which may easily lead to a model overfitting the data and becoming unnecessarily oscillating, thus corresponding poorly to the underlying function.

Unlike in the case of interpolation, there exist various possibilities to approximate a set of noisy function values by means of radial basis functions. A suitable choice essentially depends on the nature of the available data and the intended use of the resulting approximant. A technique that is frequently employed is a *least-squares approximation*, see, e.g., Buhmann [3], Chapter 8, or Iske [16], Section 3.10, where approximants of the generic form (4) are considered for a reduced numbered of pairwise distinct centres $\{\tilde{x}_j\}_{j=1}^{\tilde{n}} \subset \mathcal {X}$, $\tilde{n} + {\widetilde{m}}< n$, which usually coincide with some of the sample points $x_1,\ldots ,x_n$, but may also be different. This form is then used to obtain an optimal approximant $s \in \tilde{\mathcal {A}}_{\phi ,m}(\mathcal {X})$ by solving

17 $$\begin{aligned} \min _{s \in \tilde{\mathcal {A}}_{\phi ,m}(\mathcal {X})} \sum _{i=1}^n w_i \big ( s(x_i) - \hat{f}^{(n)} (x_i)\big )^2, \end{aligned}$$

where $\tilde{\mathcal {A}}_{\phi ,m}(\mathcal {X})$ denotes the corresponding linear function space and $w_1,\ldots ,w_n$ are positive weights to take care of potential heteroscedasticity in the data. Due to the side conditions in $\tilde{\mathcal {A}}_{\phi ,m}(\mathcal {X})$, problem (17) constitutes a linear least-squares problem with equality constraints, which can be solved uniquely via a linear system if the set of centres $\{\tilde{x}_1,\ldots ,\tilde{x}_{\tilde{n}}\}$ is $\mathcal {P}_m^d$-unisolvent and forms a subset of the sample points, see, e.g., Iske [16], Theorem 17. A least-squares approach may notably reduce the complexity of constructing an approximant if $\tilde{n} \ll n$. However, the main drawback then lies in choosing a suitable set of centres which defines both the smoothness of an approximant and its closeness to the data. This ambiguity makes it difficult to incorporate the technique into a response surface method where new points are added iteratively, as argued, for instance, by Žilinskas [51].

An approach that explicitly allows to include both the semi-norm as a measure of smoothness and the availability of error bounds into the construction of a radial basis function approximant is known as *relaxed interpolation*, see, e.g., Schaback and Wendland [38], Section 3. Specifically, requiring an approximant to be as smooth as possible but such that it deviates at the sampled points $x_i$ from the observed values $\hat{f}^{(n)}(x_i)$ by at most $\epsilon _i^{(n)}$, an optimal approximant $s \in \mathcal {A}_{\phi , m}(\mathcal {X})$ is found by solving

18 $$\begin{aligned} \begin{aligned}&\min _{s \in \mathcal {A}_{\phi , m}(\mathcal {X})}{} & {} \Vert s\Vert _\phi ^2 \\&\text {s.t.}{} & {} \big |w_i \big ( s(x_i) - \hat{f}^{(n)}(x_i) \big )\big | \le \epsilon _i^{(n)}, \quad i=1,\ldots ,n. \end{aligned} \end{aligned}$$

By definition of the semi-norm and the side conditions in $\mathcal {A}_{\phi , m} (\mathcal {X})$, problem (18) presents a convex quadratic programme with both equality and inequality constraints, which can be solved uniquely if the set of points $\{x_1,\ldots ,x_n\}$ is assumed to be $\mathcal {P}_m^d$-unisolvent. Note that, as a consequence of the involved inequality constraints, the convex quadratic programme needs to be solved; the optimal approximant can no longer be determined by solving just a linear system of equations. By applying the KKT conditions, it can be shown that the optimal approximant either interpolates the endpoints of the (potentially scaled) error bounds or the corresponding coefficient $\lambda _i$ equals zero, or both, cf. Schölkopf and Smola [39] for the related concept of support-vector machines.

The *regularised least-squares approximation*, as described, for instance, in Wendland and Rieger [50] or Wendland [48], is another approach that explicitly incorporates the semi-norm into the construction of the approximant. However, instead of imposing inequality constraints to regulate the discrepancy to the noisy function values, the closeness to the data is assessed by residual sum of squares. Consequently, an optimal approximant $s^\gamma \in \mathcal {A}_{\phi ,m}(\mathcal {X})$ is sought as the solution of

19 $$\begin{aligned} \min _{s \in \mathcal {A}_{\phi , m} (\mathcal {X})} \gamma \Vert s\Vert _\phi ^2 + \frac{1}{n} \sum _{i=1}^n w_i \big ( s(x_i) - \hat{f}^{(n)} (x_i) \big )^2, \end{aligned}$$

where the additional parameter $\gamma > 0$ is introduced to control the trade-off between the smoothness of the approximant and its closeness to the noisy function values. In particular, for large $\gamma$ we place more emphasis on minimising the bumpiness, while for small $\gamma$ the closeness to the data is enforced, yielding an interpolation of $\hat{f}^{(n)} (x_1), \ldots , \hat{f}^{(n)} (x_n)$ in case $\gamma = 0$.

Bearing in mind the constraints on the coefficients $\lambda _i$ in $\mathcal {A}_{\phi , m}(\mathcal {X})$, problem (19) comprises an equality constrained convex quadratic programme. Hence, similar to plain interpolation, the construction of an approximant can be reduced to solving a (regularised) linear system, see Theorem 1 below. In particular, this implies that errors in function values are taken into account by interpolating some perturbed noisy function values, where the magnitude of the perturbation is governed by the regularisation parameter $\gamma$. Moreover, the parameter $\gamma$ has a clear and intuitive interpretation, which facilitates its determination by means of the available error bounds (2) or (3) and also allows for a convenient application of Jones’ technique to determine new evaluation points, cf. Sect. 4. Consequently, regularised least-squares approximation seems to provide the most suitable approach for an extension of Gutmann’s RBF method to noise. In what follows, we will make use of the following result.

### Theorem 1

Let $\phi$ be a conditionally positive definite radial basis function of order $m$, and assume that a $\mathcal {P}_m^d$-unisolvent set of points $\{x_1,\ldots ,x_n\} \subset \mathcal {X}$ with corresponding noisy function values $\hat{f}^{(n)} (x_1), \ldots , \hat{f}^{(n)} (x_n)$ is given. Then, for any $\gamma >0$, the approximant $s^\gamma _n \in \mathcal {A}_{\phi , m}(\mathcal {X})$ whose coefficients are determined by the linear system

20 $$\begin{aligned} \begin{pmatrix} \varPhi + n \gamma W^{-1} &{} P \\ P^\top &{} 0 \end{pmatrix} \begin{pmatrix} \lambda \\ c \end{pmatrix} = \begin{pmatrix} \widehat{F}^{(n)} \\ 0 \end{pmatrix}, \end{aligned}$$

where $W = {\text {diag}}(w_1,\ldots ,w_n)$ and $\widehat{F}^{(n)} = (\hat{f}^{(n)} (x_1), \ldots , \hat{f}^{(n)} (x_n))^\top$, is the unique element of $\mathcal {A}_{\phi , m}(\mathcal {X})$ that solves the regularised least-squares approximation problem (19).

### Proof

Let $\gamma > 0$ be fixed, and observe that problem (19) can be rewritten as

21 $$\begin{aligned} \min _{(\lambda ,c)^\top \in \mathbb {R}^{n + {\widetilde{m}}}} n \gamma \lambda ^\top \varPhi \lambda + \big \Vert W^{1/2} (\varPhi \lambda + P c - \widehat{F}^{(n)})\big \Vert _2^2 \qquad \text {s.t.} \quad P^\top \lambda = 0. \end{aligned}$$

By the conditional positive definiteness of $\phi$, problem (21) is strictly convex. Hence, a unique solution exists if the set of sample points $\{x_1,\ldots ,x_n\}$ is $\mathcal {P}_m^d$-unisolvent, guaranteeing that the matrix $P^\top$ has full row rank. Applying the KKT conditions to (21) provides the linear equations

22 $$\begin{aligned} (n \gamma \varPhi + \varPhi ^\top W \varPhi ) \lambda + \varPhi ^\top W P c + P \upsilon&= \varPhi ^\top W \widehat{F}^{(n)} \end{aligned}$$

23 $$\begin{aligned} P^\top W (\varPhi \lambda + P c)&= P^\top W \widehat{F}^{(n)} \end{aligned}$$

24 $$\begin{aligned} P^\top \lambda&= 0 , \end{aligned}$$

where $\upsilon \in \mathbb {R}^{\widetilde{m}}$ denotes the Lagrange multiplier for the constraint $P^\top \lambda = 0$. Since $\phi$ is conditionally positive definite, the matrix $\varPhi$ is invertible for any $\lambda \in \mathbb {R}^n \backslash \{0\}$ satisfying (24). Thus, multiplying Eq. (22) by $(\varPhi W)^{-1}$ simplifies to

$$\begin{aligned} (\varPhi + n \gamma W^{-1}) \lambda + P c + (\varPhi ^\top W)^{-1} P \upsilon = \widehat{F}^{(n)}, \end{aligned}$$

which, by substituting into Eq. (23), yields $P^\top \varPhi ^{-1} P \upsilon = 0$. However, since $\{x_1,\ldots ,x_n\}$ is $\mathcal {P}_m^d$-unisolvent, the latter implies $\upsilon =0$, such that we obtain the stated linear system (20). $\square$

Note that the linear system (20) remains uniquely solvable if new points are added, distinct from already sampled points.

## A radial basis function method for noisy objective functions

In this section, we describe our novel RBF method for minimising a noisy objective function $\hat{f}:\mathcal {X}\rightarrow \mathbb {R}$ on a compact set $\mathcal {X}$, which proceeds similar to Algorithm 1 but uses a regularised least-squares approach to construct approximating response surfaces and determine new evaluation points.

Let $\phi$ be a conditionally positive definite radial basis function of order $m$ and $\mathcal {P}_m^d$ be the space of polynomials of degree at most $m-1$ with basis $\{p_j\}_{j=1}^{\widetilde{m}}$. Assume that the initially sampled points $x_1,\ldots ,x_{n_0}$ form a $\mathcal {P}_m^d$-unisolvent set and that error bounds $\epsilon _1^{(n_0)},\ldots ,\epsilon _{n_0}^{(n_0)}$ and positive weights $w_1,\ldots ,w_{n_0}$ are available for the corresponding noisy function values $\hat{f}^{(n_0)} (x_1), \ldots , \hat{f}^{(n_0)} (x_{n_0})$. For $n \ge n_0$, a general iteration, consisting of the construction of an approximant and the determination of a new evaluation point by a suitably chosen target value, can then be described as follows, cf. Sect. 4.4 for a compact description of the full algorithm.

### Construction of response surface

For given data $(x_1,\hat{f}^{(n)} (x_1)), \ldots , (x_n,\hat{f}^{(n)} (x_n))$, weights $w_1,\ldots ,w_n$, and $\gamma > 0$, let us denote the unique solution of the linear system (20), cf. Theorem 1, by $( \lambda ^{(n, \gamma )}, c^{(n, \gamma )} )$ and the optimal regularised least-squares approximant to the given data from the space $\mathcal {A}_{\phi , m} (\mathcal {X})$ by

25 $$\begin{aligned} s_n^{\gamma }(x) = \sum _{i=1}^n \lambda _i^{(n, \gamma )} \phi (\Vert x - x_i\Vert _2) + p^{(n, \gamma )}(x), \quad x \in \mathbb {R}^d. \end{aligned}$$

To determine $\gamma _n$ in the *n*-th iteration, we make use of the fact that we are predominantly interested in finding a rather smooth approximant that deviates at most by the error bounds from the noisy function values to recover the underlying function *f*. Accordingly, we first observe that the smoothness of the approximant $s_n^{\gamma }$ can alternatively be characterised in terms of the parameter $\gamma$, which seems intuitively clear from formulation (19). More formally, it can be justified by the following Proposition 1, for which we define by $\mathcal {R}(P)$ the range of the polynomial basis matrix $P \in \mathbb {R}^{n \times {\widetilde{m}}}$.

#### Proposition 1

Let $\phi$ be a conditionally positive definite radial basis function of order $m$, and let $\{x_1,\ldots ,x_n\} \subset \mathcal {X}$ be a $\mathcal {P}_m^d$-unisolvent set with corresponding noisy function values $\hat{f}^{(n)} (x_1), \ldots , \hat{f}^{(n)} (x_n)$. For $\gamma >0$, let $s^\gamma \in \mathcal {A}_{\phi , m} (\mathcal {X})$ denote the unique solution to the regularised least-squares problem (19). Then, the following holds:

- $\lambda ^{(n, \gamma )}$ and $c^{(n, \gamma )}$ depend continuously on $\gamma$.
- The optimal value of (19) is concave and monotonically increasing in $\gamma$. In case
  $(\hat{f}^{(n)} (x_1),\ldots ,\hat{f}^{(n)} (x_n))^\top \notin \mathcal {R}(P)$, then the optimal value function is strictly monotonically increasing.
- For any fixed noisy function values, the term $\Vert s^\gamma \Vert _\phi$ is monotonically decreasing in $\gamma$ and $\tfrac{1}{n} \sum _{i=1}^n w_i (s^\gamma (x_i) - \hat{f}^{(n)} (x_i))^2$ is monotonically increasing in $\gamma$. If $(\hat{f}^{(n)} (x_1),\ldots ,\hat{f}^{(n)} (x_n))^\top \notin \mathcal {R}(P)$, then these terms are strictly monotonically decreasing and increasing in $\gamma$, respectively.

#### Proof

Since the associated matrix on the left-hand side of the linear system (20) is nonsingular and depends continuously on $\gamma$, so does its inverse, which establishes statement (a).

The first part of statement (b) follows directly from the affine structure of the objective function in $\gamma$. For the second part note that it holds $\Vert s^\gamma \Vert _\phi ^2 \ne 0$ due to the assumption $(\hat{f}^{(n)} (x_1),\ldots ,\hat{f}^{(n)} (x_n))^\top \notin \mathcal {R}(P)$, see Gutmann [11], p. 318.

To show (c), let $0< \gamma < \widetilde{\gamma }$ be fixed. By the optimality of the corresponding minimisers $s^\gamma , s^{\widetilde{\gamma }} \in \mathcal {A}_{\phi , m} (\mathcal {X})$, we then have

26 $$\begin{aligned} \gamma \Vert s^\gamma \Vert _\phi ^2 + \frac{1}{n} \sum _{i=1}^n w_i \big (s^\gamma (x_i) - \hat{f}^{(n)} (x_i) \big )^2 \le \gamma \Vert s^{\widetilde{\gamma }}\Vert _\phi ^2 + \frac{1}{n} \sum _{i=1}^n w_i \big (s^{\widetilde{\gamma }}(x_i) - \hat{f}^{(n)} (x_i) \big )^2, \end{aligned}$$

and

27 $$\begin{aligned} \widetilde{\gamma } \Vert s^{\widetilde{\gamma }}\Vert _\phi ^2 + \frac{1}{n} \sum _{i=1}^n w_i \big (s^{\widetilde{\gamma }}(x_i) - \hat{f}^{(n)} (x_i) \big )^2 \le \widetilde{\gamma } \Vert s^\gamma \Vert _\phi ^2 + \frac{1}{n} \sum _{i=1}^n w_i \big (s^\gamma (x_i) - \hat{f}^{(n)} (x_i) \big )^2. \end{aligned}$$

Adding both inequalities, cancelling equal terms, and rearranging yields

$$\begin{aligned} (\widetilde{\gamma } - \gamma ) \Vert s^{\widetilde{\gamma }}\Vert _\phi ^2 \le (\widetilde{\gamma } - \gamma ) \Vert s^\gamma \Vert _\phi ^2, \end{aligned}$$

such that $\Vert s^\gamma \Vert _\phi ^2$ is monotonically decreasing in $\gamma$. Moreover, it follows that

28 $$\begin{aligned} \gamma \big (\Vert s^\gamma \Vert _\phi ^2 - \Vert s^{\widetilde{\gamma }}\Vert _\phi ^2 \big ) \le \frac{1}{n} \sum _{i=1}^n w_i \big (s^{\widetilde{\gamma }}(x_i) - \hat{f}^{(n)} (x_i) \big )^2 - \frac{1}{n} \sum _{i=1}^n w_i \big (s^\gamma (x_i) - \hat{f}^{(n)} (x_i) \big )^2, \end{aligned}$$

showing that $\tfrac{1}{n} \sum _{i=1}^n w_i (s^\gamma (x_i) -\hat{f}^{(n)} (x_i))^2$ is monotonically increasing in $\gamma$.

To establish the strict monotonicity of both functions in case $(\hat{f}^{(n)} (x_1), \ldots , \hat{f}^{(n)} (x_n))^\top \notin \mathcal {R}(P)$, we start by showing that the minimisers $s^\gamma$ and $s^{\widetilde{\gamma }}$ cannot be identical for $0< \gamma < \widetilde{\gamma }$. To this end, assume $s^\gamma \equiv s^{\widetilde{\gamma }}$ and observe that the linear system (20) provides for $i=1,\ldots ,n$,

29 $$\begin{aligned} s^\gamma (x_i) - \hat{f}(x_i) = -n \gamma w_i^{-1} \lambda _i^\gamma \quad \text {and} \quad s^\gamma (x_i) - \hat{f}(x_i) = -n \widetilde{\gamma } w_i^{-1} \lambda _i^\gamma , \end{aligned}$$

where $\lambda _i^\gamma$ denotes the *i*-th coefficient of $s^\gamma$. The latter in turn yields

$$\begin{aligned} n (\widetilde{\gamma } - \gamma ) w_i^{-1} \lambda _i^\gamma = 0, \end{aligned}$$

and therefore $\lambda _i^\gamma = 0$ for $i=1\ldots ,n$. This, however, implies that $s^\gamma \in \mathcal {P}_m^d$, such that the function values $\hat{f}^{(n)} (x_1), \ldots , \hat{f}^{(n)} (x_n)$ in (29) are interpolated by a polynomial from the linear space $\mathcal {P}_m^d$, which contradicts the assumption $(\hat{f}^{(n)} (x_1), \ldots , \hat{f}^{(n)} (x_n))^\top \notin \mathcal {R}(P)$. Since $s^\gamma \ne s^{\widetilde{\gamma }}$ and the solution of (19) is unique according to Theorem 1, we even have

30 $$\begin{aligned} \gamma \Vert s^\gamma \Vert _\phi ^2 + \frac{1}{n} \sum _{i=1}^n w_i \big (s^\gamma (x_i) - \hat{f}^{(n)}(x_i) \big )^2 < \gamma \Vert s^{\widetilde{\gamma }}\Vert _\phi ^2 + \frac{1}{n} \sum _{i=1}^n w_i \big (s^{\widetilde{\gamma }}(x_i) - \hat{f}^{(n)} (x_i) \big )^2, \end{aligned}$$

i.e. < holds instead of $\le$ in (26). Adding (30) and (27) and rearranging as before immediately yields

$$\begin{aligned} (\widetilde{\gamma } - \gamma ) \Vert s^{\widetilde{\gamma }}\Vert _\phi ^2 < (\widetilde{\gamma } - \gamma ) \Vert s^\gamma \Vert _\phi ^2, \end{aligned}$$

and thus the strict monotonicity of $\Vert s^\gamma \Vert _\phi$. Finally, the strict monotonicity of $\tfrac{1}{n} \sum _{i=1}^n w_i(s^\gamma (x_i) - \hat{f}^{(n)} (x_i))^2$ follows by (28). $\square$

#### Choosing the regularisation parameter

We will see later that to show convergence of the method it suffices to choose the regularisation parameter $\gamma$ in each step in such a way that the corresponding sequence $\{ \gamma _n \}$ converges to zero quickly enough, in particular if $\gamma _n = {\textbf {o}}(1/n)$. This can easily be achieved by choosing $\{ \gamma _n \}$ to be an appropriate exogeneous sequence, e.g. $\gamma _n = 1/n^{1+\delta }$ for some $\delta > 0$.

However, depending on the noise model, much can be gained by choosing $\gamma _n$ adaptively to control the bumpiness of the approximant $s_n^{\gamma _n}$. Proposition 1 provides a corresponding framework: the parameter $\gamma _n$ can be identified uniquely under the weak assumption that $(\hat{f}^{(n)} (x_1),\ldots ,\hat{f}^{(n)} (x_n))^\top \notin \mathcal {R}(P)$ in the following way. Finding the smoothest approximant $s_n^{\gamma _n}$ such that it deviates at the considered points $x_i$ from the noisy function values $\hat{f}^{(n)}(x_i)$ by at most $\epsilon _i^{(n)}$ can be stated as the *auxiliary problem*

31 $$\begin{aligned} \begin{aligned}&\max _{\gamma \ge 0}{} & {} \gamma \\&\text {s.t.}{} & {} \big |s_n^\gamma (x_i) - \hat{f}^{(n)} (x_i)\big | \le \epsilon _i^{(n)}, \quad i=1,\ldots ,n. \end{aligned} \end{aligned}$$

Problem (31) consists of a linear objective function in one dimension, which is subject to *n* nonlinear inequality constraints. Since $s_n^{\gamma }$ converges to the interpolant of $\hat{f}^{(n)} (x_1), \ldots , \hat{f}^{(n)} (x_n)$ for $\gamma \rightarrow 0$, as can be read off from the regularised system (20), a feasible solution to problem (31) exists. However, unlike the sum-of-squares function, the individual constraints are potentially non-monotonic in $\gamma$, and each evaluation of the constraints requires to solve the linear system (20). This renders the problem difficult to solve and unnecessarily time-consuming. Thus, as $\gamma _n$ is readjusted in each iteration upon the addition of a new point, searching for an approximate solution is sufficient. Preliminary numerical experiments indicate that appropriate values of $\gamma _n$ can be obtained by an efficient backtracking strategy, which starts with a large enough $\gamma _n$ and successively decreases this value until all constraints of (31) are met for the first time.

### Determination of next evaluation point

To determine the next point of evaluation $x_{n+1}$, we continue similar to Jones’ technique and assume that a noise-free target value $f_n^*$ has been chosen. Let $\gamma _n > 0$ be chosen appropriately. Then, let $x_{n+1}$ be the point $y \in \mathcal {X}\backslash \{x_1,\ldots ,x_n\}$ such that the augmented approximant $s_y^{\gamma _n} \in \mathcal {A}_{\phi , m} (\mathcal {X})$ minimises the regularised least-squares criterion to previous sample points and interpolates $f_n^*$ at the new *y*. In formal terms, we thus require that $s_y^{\gamma _n}$ solves

32 $$\begin{aligned} \min _{s \in \mathcal {A}_{\phi , m} (\mathcal {X})} \gamma _n \Vert s\Vert _\phi ^2 + \frac{1}{n} \sum _{i=1}^n w_i \big ( s(x_i) - \hat{f}^{(n)} (x_i) \big )^2 \qquad \text {s.t.} \quad s(y) = f_n^*, \end{aligned}$$

which is a strictly convex optimisation problem on $\mathcal {A}_{\phi , m} (\mathcal {X})$ and thus admits a unique solution, cf. Theorem 1. Note that (32) is a penalised version of problem (9), in the sense that we relax *n* interpolation conditions by adding quadratic penalty terms to the objective.

To simplify problem (32) in terms of the sought new point $y \in \mathcal {X}\backslash \{x_1,\ldots ,x_n\}$, we first rewrite the augmented approximant $s_y^{\gamma _n}$ according to

33 $$\begin{aligned} s_y^{\gamma _n}(x) = s_n^{\gamma _n}(x) + \big [ f_n^*- s_n^{\gamma _n}(y)\big ] \, l_n^{\gamma _n}(y,x), \quad x \in \mathbb {R}^d, \end{aligned}$$

where $l_n^{\gamma _n}(y,{\hspace{1.111pt}\cdot \hspace{1.111pt}}) \in \mathcal {A}_{\phi , m} (\mathcal {X})$ is the radial basis function approximant that solves the constrained regularised least-squares problem

34 $$\begin{aligned} \min _{l(y,{\hspace{1.111pt}\cdot \hspace{1.111pt}}) \in \mathcal {A}_{\phi , m} (\mathcal {X})} \gamma _n \Vert l(y,{\hspace{1.111pt}\cdot \hspace{1.111pt}})\Vert _\phi ^2 + \frac{1}{n} \sum _{i=1}^n w_i \big ( l(y,x_i) \big )^2 \qquad \text {s.t.} \quad l(y,y) = 1. \end{aligned}$$

Representation (33) is valid since for any $y \in \mathcal {X}\backslash \{x_1,\ldots ,x_n\}$ both $s_n^{\gamma _n}$ and $l_n^{\gamma _n}(y,{\hspace{1.111pt}\cdot \hspace{1.111pt}})$ are uniquely defined as solutions to the problems (19) and (34), respectively. Hence, the right-hand side of (33) is a unique well-defined element of $\mathcal {A}_{\phi , m} (\mathcal {X})$, and it also satisfies the interpolation constraint in problem (32). Moreover, similar to Theorem 1, it can be shown that the approximating function $l_n^{\gamma _n}(y,{\hspace{1.111pt}\cdot \hspace{1.111pt}})$ has the form

$$\begin{aligned} l_n^{\gamma _n}(y,x) = \sum _{i=1}^n \alpha _i(y) \phi (\Vert x-x_i\Vert _2) + \beta (y) \phi (\Vert x-y\Vert _2) + \sum _{j=1}^{{\widetilde{m}}} b_j(y) p_j(x), \quad x \in \mathbb {R}^d, \end{aligned}$$

where the coefficients^1^$\alpha (y) = (\alpha _1(y),\ldots ,\alpha _n(y))^\top \in \mathbb {R}^n$, $\beta (y) \in \mathbb {R}$, and $b(y) = (b_1(y),\ldots ,b_{{\widetilde{m}}}(y))^\top \in \mathbb {R}^{{\widetilde{m}}}$ are defined by the linear system

35 $$\begin{aligned} \begin{pmatrix} \varPhi + n \gamma _n W^{-1} &{}\quad u_n(y) &{}\quad P \\ u_n(y)^\top &{}\quad \phi (0) &{}\quad \pi (y)^\top \\ P^\top &{}\quad \pi (y) &{}\quad 0 \end{pmatrix} \begin{pmatrix} \alpha (y) \\ \beta (y) \\ b(y) \end{pmatrix} = \begin{pmatrix} 0 \\ 1 \\ 0 \end{pmatrix}, \end{aligned}$$

for the matrices $\varPhi \in \mathbb {R}^{n \times n}$, $P \in \mathbb {R}^{n \times {\widetilde{m}}}$, and $W \in \mathbb {R}^{n \times n}$ introduced before, and the corresponding vectors $u_n(y) = (\phi (\Vert x_1-y\Vert _2),\ldots ,\phi (\Vert x_n-y\Vert _2))^\top \in \mathbb {R}^n$ and $\pi (y) = (p_1(y),\ldots ,p_{{\widetilde{m}}}(y))^\top \in \mathbb {R}^{\widetilde{m}}$. Note that (35) can be seen as a regularised version of (12), where positive entries have been added to the diagonal of the interpolation matrix $\varPhi$.

By inserting representation (33) into the objective function of (32), the latter can then be reformulated as

36 $$\begin{aligned}&\gamma _n \Vert s_y^{\gamma _n}\Vert _\phi ^2 + \frac{1}{n} \sum _{i=1}^n w_i \big ( s_y^{\gamma _n}(x_i) - \hat{f}^{(n)} (x_i) \big )^2 \nonumber \\&\quad = \gamma _n \Big ( \Vert s_n^{\gamma _n}\Vert _\phi ^2 + 2 \big [ f_n^*- s_n^{\gamma _n}(y) \big ] \langle s_n^{\gamma _n}, l_n^{\gamma _n}(y,{\hspace{1.111pt}\cdot \hspace{1.111pt}}) \rangle _\phi + \big [ f_n^*- s_n^{\gamma _n}(y) \big ]^2 \Vert l_n^{\gamma _n}(y,{\hspace{1.111pt}\cdot \hspace{1.111pt}})\Vert _\phi ^2 \Big ) \nonumber \\&\qquad \qquad + \frac{1}{n} \sum _{i=1}^n w_i \Big ( \big ( s_n^{\gamma _n}(x_i) - \hat{f}^{(n)} (x_i) \big )^2 + \big [ f_n^*- s_n^{\gamma _n}(y) \big ]^2 \big ( l_n^{\gamma _n}(y,x_i) \big )^2 \nonumber \\&\qquad \qquad + 2 \big ( s_n^{\gamma _n}(x_i) - \hat{f}^{(n)} (x_i) \big ) \big [f_n^*- s_n^{\gamma _n}(y) \big ] l_n^{\gamma _n}(y,x_i) \Big ) \nonumber \\&\quad = \gamma _n \Vert s_n^{\gamma _n}\Vert _\phi ^2 + \frac{1}{n}\sum _{i=1}^n w_i \big (s_n^{\gamma _n}(x_i) - \hat{f}^{(n)} (x_i) \big )^2 \nonumber \\&\qquad \qquad + \big [ f_n^*- s_n^{\gamma _n}(y)\big ]^2 \Big ( \gamma _n \Vert \, l_n^{\gamma _n}(y,{\hspace{1.111pt}\cdot \hspace{1.111pt}})\Vert _\phi ^2 + \frac{1}{n} \sum _{i=1}^n w_i \bigg ( l_n^{\gamma _n}(y,x_i) \big )^2 \bigg ) , \end{aligned}$$

where the last equation holds by definition of the semi-inner product (6) and the relation $s_n^{\gamma _n}(x_i) - \hat{f}^{(n)} (x_i) = - n \gamma _n w_i^{-1} \lambda _i$, $i=1,\ldots ,n$, due to (20), which both together yield

$$\begin{aligned} \langle s_n^{\gamma _n}, l_n^{\gamma _n}(y,{\hspace{1.111pt}\cdot \hspace{1.111pt}}) \rangle _\phi&= \sum _{i=1}^n \lambda _i l_n^{\gamma _n}(y,x_i) \\&= -\frac{1}{n \gamma _n} \sum _{i=1}^n w_i \big ( s_n^{\gamma _n}(x_i) - \hat{f}^{(n)} (x_i) \big ) l_n^{\gamma _n}(y,x_i). \end{aligned}$$

Now, the first two terms on the right-hand side of Eq. (36) are independent of *y* and correspond to the objective function for constructing the approximant $s_n^{\gamma _n}$, cf. problem (19). To find the new point *y*, it thus suffices to consider the last term in (36). However, by the semi-inner product (6) and the linear system (35), implying $l_n^{\gamma _n}(y,x_i) = - n \gamma _n w_i^{-1} \alpha _i(y)$ for $i=1,\ldots ,n$, it holds

$$\begin{aligned}&\gamma _n \Vert l_n^{\gamma _n}(y,{\hspace{1.111pt}\cdot \hspace{1.111pt}})\Vert _\phi ^2 + \frac{1}{n } \sum _{i=1}^n w_i \big (l_n^{\gamma _n}(y,x_i) \big )^2 \\&\qquad = \gamma _n \left( \sum _{i=1}^n \alpha _i(y) l_n^{\gamma _n}(y,x_i) + \beta (y) l_n^{\gamma _n}(y,y) \right) + \frac{1}{n}\sum _{i=1}^n w_i \big ( l_n^{\gamma _n}(y,x_i) \big )^2 \\&\qquad = \gamma _n \beta (y). \end{aligned}$$

Therefore, we can conclude that solving the required problem (32) is equivalent to minimising the nonnegative function

37 $$\begin{aligned} g_n^{\gamma _n}(y) := \mu _n^{\gamma _n}(y) \big [f_n^*- s_n^{\gamma _n}(y)\big ]^2, \qquad y \in \mathcal {X}\backslash \{x_1,\ldots ,x_n\} \end{aligned}$$

with respect to *y*, where the function $\mu _n^{\gamma _n}: \mathcal {X}\backslash \{x_1,\ldots ,x_n\} \rightarrow \mathbb {R}$ is defined for $\gamma _n>0$ by

38 $$\begin{aligned} \mu _n^{\gamma _n}(y) := \Vert l_n^{\gamma _n}(y,{\hspace{1.111pt}\cdot \hspace{1.111pt}})\Vert _\phi ^2 + \frac{1}{n \gamma _n} \sum _{i=1}^n w_i \big ( l_n^{\gamma _n}(y,x_i) \big )^2 = \beta (y). \end{aligned}$$

Note the resemblance of the functions $g_n^{\gamma _n}$ and $\mu _n^{\gamma _n}$ to their deterministic counterparts (14) and (15), respectively. In particular, since $l_n^{\gamma _n}(y,{\hspace{1.111pt}\cdot \hspace{1.111pt}})$ is well-defined for $y \in \mathcal {X}\backslash \{x_1,\ldots ,x_n\}$, so are both functions $g_n^{\gamma _n}$ and $\mu _n^{\gamma _n}$.

#### Remark 4

By the same argument as given in Remark 1, definition (38) implies that the function $\mu _n^{\gamma _n}$ is positive on $\mathcal {X}\backslash \{x_1,\ldots ,x_n\}$.

Moreover, by definition (38) and Cramer’s rule to solve the linear system (35), we have

$$\begin{aligned} \mu _n^{\gamma _n}(y) = \frac{\det {A}_n^{\gamma _n}}{\det {A_n^{\gamma _n}(y)}}, \qquad y \in \mathcal {X}\backslash \{x_1,\ldots ,x_n\}, \end{aligned}$$

where $A_n^{\gamma _n}$ and $A_n^{\gamma _n}(y)$ denote the nonsingular matrices on the left-hand sides of the linear systems (20) and (35), respectively, that is

39 $$\begin{aligned} A_n^{\gamma _n} := \begin{pmatrix} \varPhi + n \gamma _n W^{-1} &{}\quad P \\ P^\top &{}\quad 0 \end{pmatrix} \quad \text {and} \quad A_n^{\gamma _n}(y) := \begin{pmatrix} \varPhi + n \gamma _n W^{-1} &{}\quad u_n(y) &{}\quad P \\ u_n(y)^\top &{}\quad \phi (0) &{}\quad \pi (y)^\top \\ P^\top &{}\quad \pi (y) &{}\quad 0 \end{pmatrix}. \end{aligned}$$

Since $\det A_n^{\gamma _n}$ is a nonzero constant and $\lim _{y \rightarrow x_i} \det A_n^{\gamma _n}(y) \ne 0$ $(i \in \{1,\ldots ,n\})$ by the positivity of $v_n^{\gamma _n}$, it thus holds

$$\begin{aligned} \lim _{y \rightarrow x_i} \mu _n^{\gamma _n}(y) < \infty , \qquad i=1,\ldots ,n. \end{aligned}$$

Hence, even though $\mu _n^{\gamma _n}$ is not defined at the sample points $x_1,\ldots ,x_n$, it can be continuously extended at these points by the positive and finite values

40 $$\begin{aligned} \mu _n^{\gamma _n}(x_i) = \frac{\det {A}_n^{\gamma _n}}{\det {A_n^{\gamma _n}(x_i)}}, \qquad i=1,\ldots ,n, \end{aligned}$$

due to the continuity of the determinant.

In an analogous manner to Gutmann [12], Proposition 4.12 (cf. Remark 3), the function $\mu _n^{\gamma _n}$ can be rewritten according to the following proposition.

#### Proposition 2

For $\gamma _n>0$, the function $v_n^{\gamma _n}$ defined by

$$\begin{aligned} v_n^{\gamma _n}(y) := \Bigg [ \phi (0) - \begin{pmatrix} u_n(y) \\ \pi (y) \end{pmatrix}^\top \begin{pmatrix} \varPhi + n \gamma _n W^{-1} &{}\quad P \\ P^\top &{}\quad 0 \end{pmatrix}^{-1} \begin{pmatrix} u_n(y) \\ \pi (y) \end{pmatrix} \Bigg ], \qquad y \in \mathbb {R}^d, \end{aligned}$$

is identical to $1/ \mu _n^{\gamma _n}$ on $y \in \mathbb {R}^d\backslash \{x_1,\ldots ,x_n\}$. Moreover, $v_n^{\gamma _n}$ can be continuously extended at the sample points $x_1,\ldots ,x_n$ by the finite values $v_n^{\gamma _n}(x_i) = 1/\mu _n^{\gamma _n}(x_i)$, $i=1,\ldots ,n$.

#### Proof

For any $y \in \mathbb {R}^d \backslash \{x_1,\ldots ,x_n\}$, the proof follows in a straightforward manner by using the definition of $\mu _n(y)$ in (38) and solving the equations in the linear system (35) for the coefficient $\beta (y)$ by rearranging and applying the Schur complement of the invertible block $A_n^{\gamma _n}$ in the matrix $A_n^{\gamma _n}(y)$. $\square$

Since the function $v_n^{\gamma _n}$ is positive and finite on $\mathcal {X}$ for $\gamma _n>0$, it can be understood as a measure of uncertainty in the approximating model $s_n^{\gamma _n}$ to *f*. In particular, the error $v_n^{\gamma _n}(y)$ at any *y* is influenced by the distance to the sample points $x_1,\ldots ,x_n$ as well as the inherent noise resulting from inexact function values, which is most notably reflected by the fact that $v_n^{\gamma _n}(x_i) > 0$ for $i=1,\ldots ,n$.

Finally, based on Proposition 2, the function $h_n^{\gamma _n}$ defined by

$$\begin{aligned} h_n^{\gamma _n}(y) := \frac{v_n^{\gamma _n}(y)}{\big [s_n^{\gamma _n}(y) - f_n^*\big ]^2}, \qquad y \in \mathbb {R}^d, \end{aligned}$$

can be shown to be identical to $1/g_n^{\gamma _n}$ on $y \in \mathbb {R}^d\backslash \{x_1,\ldots ,x_n\}$, with continuously extended values $h_n^{\gamma _n}(x_i) = 1/g_n^{\gamma _n}(x_i)$, $i=1,\ldots ,n$.

### Choice of target value

As the target value $f_n^*$ has the same functionality as in Algorithm 1, its choice determines the location of the next evaluation point $x_{n+1}$, minimising $g_n^{\gamma _n}$ on $\mathcal {X}\backslash \{x_1,\ldots ,x_n\}$ for fixed $\gamma _n > 0$, in a similar fashion to Algorithm 1. For $f_n^*< \min _{y \in \mathcal {X}} s_n^{\gamma _n}(y)$ we usually interpret $f_n^*$ as the next target function value, i.e. we want to compute an argument $x_{n+1}$ with objective function value close to $f_n^*$. Unfortunately, however, it remains unclear at this point whether a choice $f_n^*< \min _{y \in \mathcal {X}} s_n^{\gamma _n}(y)$ is also sufficient to guarantee that a global minimiser of $g_n^{\gamma _n}$ on $\mathcal {X}\backslash \{x_1,\ldots ,x_n\}$ exists, i.e. that a global minimiser of $g_n^{\gamma _n}$ over $\mathcal {X}$ does not coincide with any of the sample points $x_1,\ldots ,x_n$, as in the case of interpolation, or whether a further condition is required. The main issue here is due to the fact that $g_n^{\gamma _n}$ is continuously extendable at the points $x_i$, $i=1,\ldots ,n$, by a finite value, cf. Eq. (40), which then implies that $g_n^{\gamma _n}(y)$ does not tend to infinity anymore as *y* approaches any $x_i$.

In any case, though, note that for an admissible choice $f_n^*< \min _{y \in \mathcal {X}} s_n^{\gamma _n}(y)$, we may draw the same conclusions for $g_n^{\gamma _n}$ as for $g_n$ in that, for $f_n^*= -\infty$, the minimisation of $g_n^{\gamma _n}$ on $\mathcal {X}\backslash \{x_1,\ldots ,x_n\}$ reduces to minimising $\mu _n^{\gamma _n}$ on $\mathcal {X}\backslash \{x_1,\ldots ,x_n\}$. Moreover, due to the identities $v_n^{\gamma _n} = 1/\mu _n^{\gamma _n}$ and $h_n^{\gamma _n} = 1/g_n^{\gamma _n}$, as established in the previous subsection, the minimisers of $\mu _n^{\gamma _n}$ and $g_n^{\gamma _n}$ correspond to the maximisers of $v_n^{\gamma _n}$ and $h_n^{\gamma _n}$ on $\mathcal {X}\backslash \{x_1,\ldots ,x_n\}$, respectively. Hence, if $-\infty< f_n^*< \min _{y \in \mathcal {X}} s_n^{\gamma _n}(y)$, we can equivalently maximise the utility function $h_n^{\gamma _n}$ and, if $f_n^*= -\infty$, the respective function $v_n^{\gamma _n}$ on $\mathcal {X}\backslash \{x_1,\ldots ,x_n\}$.

In case we end up in a situation in which one of the $x_i$ is a global minimiser of $g_n^{\gamma _n}$, we would revisit $x_{n+1} = x_i$ instead of finding a new point. In this case, there are various ways ahead, bearing in mind that we want to construct a sequence $\{x_n\}$ that is dense in $\mathcal {X}$. We could thus consider, e.g., a Delaunay triangulation induced by the $x_1, \ldots , x_n$ and then choose $x_{n+1}$ as the center point of the largest simplex in the triangulation, or update $f_n^*$ to a smaller value and repeat the iteration. Since this is not of importance in the convergence proofs and to keep the exposition succinct, we will not describe how to handle this case when stating our algorithm in the next subsection.

### Summary of the RBF method for noisy objective functions

In summary, the RBF method for minimising a noisy objective function $\hat{f}:\mathcal {X}\rightarrow \mathbb {R}$ on a compact set $\mathcal {X}$ can be formulated by the following algorithm.

#### Algorithm 2

(RBF Method for Noisy Objective Functions).

0. **Initial step:**
  Choose a conditionally positive definite radial basis function $\phi$ of order $m$.
  Generate a $\mathcal {P}_m^d$-unisolvent set of points $\{x_1,\ldots ,x_{n_0}\} \subset \mathcal {X}$.
  Evaluate $\hat{f}$ at the points $x_1,\ldots ,x_{n_0}$, resulting in $\hat{f}^{(n_0)}(x_1),\ldots ,\hat{f}^{(n_0)}(x_{n_0})$.
  Choose $x^{(n_0)} \in \mathop {{\hbox {arg min}}}\limits _{1\le i \le n_0} \Big \{\hat{f}^{(n_0)}(x_i) + \epsilon _i^{(n_0)} \Big \}$, and set $n=n_0$.
1. **Iteration step:**
  **while** $$n \le n^{\max }$$ **do**
    Choose $$\gamma _n > 0$$.
    Construct the approximant $s_n^{\gamma _n} \in \mathcal {A}_{\phi , m} (\mathcal {X})$ solving
      $$\begin{aligned} \min _{s \in \mathcal {A}_{\phi , m} (\mathcal {X})} \, \gamma _n \Vert s\Vert _\phi ^2 + \frac{1}{n} \sum _{i=1}^n w_i \big (s(x_i) - \hat{f}^{(n)} (x_i)\big )^2 . \end{aligned}$$
    Choose an admissible target value $f_n^*\in \big [-\infty , \, \min _{y \in \mathcal {X}} s_n^{\gamma _n}(y) \big ]$.
    Determine $x_{n+1}$, which is the value of *y* that solves
      $$\begin{aligned} \min _{y \in \mathcal {X}\backslash \{x_1,\ldots ,x_n\}} \mu _n^{\gamma _n}(y) \big [f_n^*- s_n^{\gamma _n}(y) \big ]^2. \end{aligned}$$
    For $i = 1,\ldots ,n$, update the evaluation $\hat{f}^{(n)} (x_i)$ to $\hat{f}^{(n+1)} (x_i )$.
    Evaluate $\hat{f}$ at $x_{n+1}$, resulting in $\hat{f}^{(n+1)}(x_{n+1})$.
    Choose $$x^{(n+1)} \in \mathop {{\hbox {arg min}}}\limits _{1\le i\le n+1} \Big \{ \hat{f}^{(n+1)}(x_i) + \epsilon _i^{(n+1)} \Big \}$$.
    Set $$n=n+1$$.
  **end while**

## Convergence of method

As Gutmann’s original method, our RBF method for noisy objective functions is a purely deterministic sequential sampling algorithm. For a given set of noisy function values, the construction of an approximant and the subsequent selection of a new evaluation point is carried out independently of any source of randomness. To show convergence of the method to the global minimum of any continuous function *f* by means of noisy objective function values $\hat{f}^{(n)} (x_i)$, our main task is thus to establish the density of the sequence of generated iterates $\{x_n\}$ in $\mathcal {X}$, cf. Törn and Žilinskas [43], Theorem 1.3. We can therefore state the following obvious theorem for the convergence of Algorithm 2.

### Theorem 2

Let *f* be a continuous function on the compact set $\mathcal {X}$ with minimum function value $f^*$. Suppose $\epsilon _n \rightarrow 0$ for $n \rightarrow \infty$ in the case of fixed noise and $\max _{1 \le i \le n} \epsilon _i^{(n)} \rightarrow 0$ for $n \rightarrow \infty$ in the case of vanishing iterative noise. Then, Algorithm 2 provides a sequence $\{ x^{(n)} \}$ with $\lim _{n \rightarrow \infty } \hat{f}^{(n)} (x^{(n)}) = \lim _{n \rightarrow \infty } f (x^{(n)}) = f^*$ if it generates a sequence of points $\{x_n\}$ that is dense in $\mathcal {X}$.

As it turns out in the below, a key result that allows to establish the density of the sequence of generated points $\{x_n\}$ is the relationship

$$\begin{aligned} \lim _{n \rightarrow \infty } n \gamma _n = 0, \end{aligned}$$

i.e. $\gamma _n = {\textbf {o}}(1/n)$. To this end, we first address the influence of the error bounds $\{\epsilon _i^{(n)}\}$ on the sequence $\{n \gamma _n\}$, provided that $\gamma _n$ is chosen in each iteration according to the auxiliary problem (31). We then present several convergence results, followed by a proof of convergence of the main statement.

### Assumptions on error bounds

One possibility for establishing density of the iterates $x_1, \ldots , x_n$ is to resort to the available convergence results of Gutmann’s method (see the supplementary Section A for a brief summary of the main results), and show that these pertain if the exact function values $f(x_i)$ are replaced by the noisy observations $\hat{f}^{(n)} (x_i)$ ($1 \le i \le n$). An indispensable assumption is thus that the involved level of noise decreases to zero over the course of the optimisation. As one may already conjecture from the construction of regularised least-squares approximants through (20), this will be required to adopt Gutmann’s proof of convergence for noisy function values. Nevertheless, in the vanishing iterative noise model, for a natural choice of $\gamma _n$, the sequence $\{ n \gamma _n\}$ converges to zero if we require that $\epsilon _i^{(n)} \rightarrow 0$ fast enough as $n \rightarrow \infty$, as the following Theorem 3 shows.

#### Theorem 3

Let $\phi$ be a conditionally positive definite radial basis function of order $m$, and let $\{x_1,\ldots ,x_n\} \subset \mathcal {X}$ be a $\mathcal {P}_m^d$-unisolvent set. Let $s_n^{\gamma _n} \in \mathcal {A}_{\phi , m} (\mathcal {X})$ denote the unique optimal solution of the regularised least-squares problem (19), where the regularisation parameter $\gamma _n > 0$ solves

41 $$\begin{aligned} \begin{aligned}&\max _{\gamma \ge 0}&\gamma \\&\text {s.t.}&\big |s_n^\gamma (x_i) - \hat{f}^{(n)} (x_i)\big | \le \epsilon _i^{(n)}, \quad i=1,\ldots ,n, \end{aligned} \end{aligned}$$

for some positive error bounds $\epsilon _i^{(n)}$ and assume $\Vert s_n^{\gamma _n} \Vert _{\phi } \ge \bar{s} > 0$ for sufficiently large *n*:

1. Assume that the sequence $\{ S_n \}$ with
  $$\begin{aligned} S_n := \inf _{\begin{array}{c} {s \in \mathcal {A}_{\phi , m}}\\ {\Vert s\Vert _{\phi } = 0} \end{array}} \sum _{i=1}^n w_i \left( s(x_i)- \hat{f}^{(n)} (x_i) \right) ^2 \end{aligned}$$
  is bounded. Then, $\gamma _n = {\textbf {O}}(1/n)$.
2. Assume that the sequence $\{ w_i \}$ is bounded above, i.e. $w_i \le \bar{w}$. Then we have that
  $$\begin{aligned} \gamma _n^2 \le \frac{\bar{\phi }\bar{w}}{\bar{s}^2} \frac{1}{n} \sum _{i=1}^n w_i (\epsilon _i^{(n)})^2 \end{aligned}$$
  holds, where $$\bar{\phi } = \max _{u, v \in \mathcal {X}}\phi (\Vert u-v\Vert _2).$$
3. Assume that $\varPhi _{i,j} \ge 0$ for all *i*, *j*. Then we have that
  $$\begin{aligned} \gamma _n^2 \le \frac{\bar{\phi }}{\bar{s}^2} \frac{1}{n} \sum _{i=1}^n (w_i \epsilon _i^{(n)})^2 \end{aligned}$$
  holds.

In the second or third case, it follows that $\gamma _n = {\textbf {o}}(1/n)$ if the errors vanish fast enough, i.e. if $\sum _{1 \le i \le n} w_i (\epsilon _i^{(n)})^2 = {\textbf {o}}(1/n)$ or $\sum _{1 \le i \le n} (w_i \epsilon _i^{(n)})^2 = {\textbf {o}}(1/n)$ holds resp.

#### Proof

1. Due to the assumption $\Vert s_n^{\gamma _n} \Vert _{\phi } \ge \bar{s} > 0$ for sufficiently large *n* we have
  $$\begin{aligned} n \gamma _n \bar{s}^2 \le n \gamma _n \Vert s_n^{\gamma _n} \Vert _{\phi }^2 \le n \gamma _n \Vert s_n^{\gamma _n} \Vert _{\phi }^2 + \sum _{i=1}^n w_i \left( s_n^{\gamma _n}(x_i) - \hat{f}^{(n)} (x_i) \right) ^2. \end{aligned}$$
  Since $s_n^{\gamma _n} \in \mathcal {A}_{\phi , m} (\mathcal {X})$ is the optimal solution of (19), we get
  $$\begin{aligned}{} & {} n \gamma _n \Vert s_n^{\gamma _n} \Vert _{\phi }^2 + \sum _{i=1}^n w_i \left( s_n^{\gamma _n}(x_i) - \hat{f}^{(n)} (x_i) \right) ^2 \le \inf _{\begin{array}{c} {s \in \mathcal {A}_{\phi , m}}{\Vert s\Vert _{\phi } = 0} \end{array}} \\{} & {} \qquad n \gamma _n \Vert s \Vert _{\phi }^2 + \sum _{i=1}^n w_i \left( s(x_i)- \hat{f}^{(n)} (x_i) \right) ^2. \end{aligned}$$
  Since the first term in this minimisation problem vanishes, this can be bounded by an $\bar{S} < + \infty$ by assumption. In summary,
  $$\begin{aligned} n \gamma _n \bar{s}^2 \le \bar{S}, \end{aligned}$$
  which proves the statement.
2. For given *n* and $\gamma > 0$, let $\lambda = \lambda ^{(n, \gamma )}$ and $c = c^{(n, \gamma )}$ denote the unique solution of the linear system (20), i.e. $\lambda$ and *c* are the coefficients of the optimal solution $s_n^\gamma$ of (19). Then, considering the first block of Eq. (20), we especially obtain
  $$\begin{aligned} \varPhi \lambda + n \gamma W^{-1} \lambda + P c = \widehat{F}^{(n)} . \end{aligned}$$
  The *i*-th row of this equation shows
  $$\begin{aligned} s_n^{\gamma } (x_i) + n \gamma w_i^{-1} \lambda _i = \hat{f}^{(n)}(x_i), \end{aligned}$$
  and thus
  42 $$\begin{aligned} \vert s_n^{\gamma } (x_i) - \hat{f}^{(n)} (x_i ) \vert = \vert n \gamma w_i^{-1} \lambda _i \vert . \end{aligned}$$
  Further, considering the matrix $\varPhi$, we can bound the maximum eigenvalue $\kappa (\varPhi )$ by the Frobenius norm of $\varPhi$ to obtain
  $$\begin{aligned} \kappa (\varPhi )^2 \le ||\varPhi ||_F^2 = \sum _{i,j} \varPhi _{i,j}^2 \le n^2 \max _{u, v \in \mathcal {X}}\phi (\Vert u-v\Vert _2)^2 = n^2 \bar{\phi }^2. \end{aligned}$$
  Hence, for sufficiently large *n*,
  43 $$\begin{aligned} \bar{s}^2 \le \Vert s_n^{\gamma _n}\Vert ^2_\phi = \lambda ^\top \varPhi \lambda \le \kappa (\varPhi ) \Vert \lambda \Vert _2^2 \le n \bar{\phi } \Vert \lambda \Vert _2^2 \end{aligned}$$
  holds for $s_n^{\gamma _n}$. Since the weights $w_i$ are bounded above by $\bar{w}$, squaring (42) for $\gamma = \gamma _n$ yields
  $$\begin{aligned} \sum _{i=1}^n w_i \left( s_n^{\gamma _n} (x_i) - \hat{f}^{(n)} (x_i ) \right) ^2 = n^2 \gamma _n^2 \sum _{i=1}^n \frac{1}{w_i} \lambda _i^2 \ge n^2 \gamma _n^2 \frac{1}{\bar{w}} \Vert \lambda \Vert _2^2. \end{aligned}$$
  Combining this with inequality (43), we obtain
  $$\begin{aligned} \bar{s}^2 \le \frac{\bar{\phi }\bar{w}}{n \gamma _n^2} \sum _{i=1}^n w_i \left( s_n^{\gamma _n} (x_i) - \hat{f}^{(n)} (x_i ) \right) ^2 \le \frac{\bar{\phi }\bar{w}}{n \gamma _n^2} \sum _{i=1}^n w_i (\epsilon _i^{(n)})^2, \end{aligned}$$
  where the last inequality follows from (41). This shows the claim.
3. Here, we proceed as before, up to Eq. (43). Combining (41) with (42) yields for $\gamma = \gamma _n$:
  $$\begin{aligned} \vert n \gamma _n \lambda _i \vert \le w_i \epsilon _i^{(n)}, \end{aligned}$$
  and, since $\varPhi$ only contains non-negative entries, we get for all *i*, *j*:
  $$\begin{aligned} \vert n^2 \gamma _n^2 \lambda _i \varPhi _{i,j} \lambda _j \vert \le w_i \epsilon _i^{(n)} \varPhi _{i,j} w_j \epsilon _j^{(n)}. \end{aligned}$$
  For notational brevity, let $\epsilon ^{(n)}:= (\epsilon ^{(n)}_1, \ldots , \epsilon ^{(n)}_n )^\top$; then
  $$ n^2 \gamma _n^2 \lambda ^\top \!\varPhi \lambda \!\le \! n^2 \gamma _n^2 \!\sum _{i,j} \vert \lambda _i \varPhi _{i,j} \lambda _j \vert \!\le \! n^2 \gamma _n^2 \!\sum _{i,j} w_i \epsilon _i^{(n)} \varPhi _{i,j} w_j \epsilon _j^{(n)} \!=\! {\epsilon ^{(n)}}^\top W \varPhi W \epsilon ^{(n)} $$
  for all $\gamma$ feasible for (41). As noted before,
  $$ \lambda ^\top \varPhi \lambda =\Vert s_n^{\gamma _n}\Vert ^2_\phi \ge \bar{s}^2, $$
  hence we get for sufficiently large *n* that
  $$ n^2 \gamma _n^2 \bar{s}^2 \le n^2 \gamma _n^2 \lambda ^\top \varPhi \lambda \le {\epsilon ^{(n)}}^\top W \varPhi W \epsilon ^{(n)} \le \kappa (\varPhi )\Vert W \epsilon ^{(n)}\Vert _2^2 \le n \bar{\phi } \Vert W \epsilon ^{(n)}\Vert _2^2, $$
  which shows the claim.

#### Remark 5

We note that Theorem 3 provides at least two cases of vanishing iterative noise, where $\gamma _n$ goes to 0 fast enough to guarantee convergence of our algorithm; roughly speaking, $\epsilon _i^{(n)}$ needs to vanish faster than 1/*n*. Further note that the assumption that $\{w_i\}$ is bounded is usually satisfied. Finally, while the assumption that $\varPhi _{i,j} \ge 0$ does not require any further assumption on $\{w_i\}$, it rules out surface spline radial basis functions of the form $r^\nu \log {r}$, where $\nu \in \mathbb {N}$ is even, cf. Table 2.

### Convergence results

Besides assuming $n\gamma _n \rightarrow 0$ as $n \rightarrow \infty$, we further require the target values $f_n^*$ to be set sufficiently low compared to the approximating surfaces $s_n^{\gamma _n}$ in order to achieve convergence of the RBF method for noisy objective functions, cf. Gutmann [12], condition (4.16) for the deterministic case. Due to the presence of noise, the critical thresholds for $f_n^*$ need to be adjusted marginally to guarantee convergence of the method in a similar fashion as Gutmann. To this end, we let, for infinitely many $n \in \mathbb {N}$, the target values $f_n^*$ satisfy

44 $$\begin{aligned} f_n^*< \min _{y \in \mathcal {X}} \Big [ s_n^{\gamma _n}(y) - \tau \Vert s_n^{\gamma _n}\Vert _\infty \big [ \varDelta _n(y) + \widetilde{w}_n^{-1/2}(y) \big ]^{\rho /2} \Big ], \end{aligned}$$

where, as in the noise-free counterpart, $\tau >0$ and $\rho \ge 0$ are constants with $\rho < 1$, for $\phi (r) = r$, and $\rho < 2$, otherwise, and $\varDelta _n$ denotes the minimum distance function

45 $$\begin{aligned} \varDelta _n(y) := \min _{1 \le i \le n} \Vert y - x_i\Vert _2, \quad y \in \mathcal {X}. \end{aligned}$$

For given $y \in \mathcal {X}$, the function $\widetilde{w}_n(y)$ gives the weight $w_i$ of the sample point $x_i$ that is closest to *y*, i.e. for $i(y) = {\hbox {arg min}}_{1\le i \le n} \Vert y-x_i\Vert _2$, we have

46 $$\begin{aligned} \widetilde{w}_n(y) := w_{i(y)}, \end{aligned}$$

with the convention that the largest *i*(*y*) is selected among the minimising indices if ${\hbox {arg min}}$ is not unique.

Finally, note that the convergence of the method is restricted to the choice of radial basis function $\phi$ as its proof requires to bound the sequence $\{\mu _n^{\gamma _n}(y)\}$ uniformly from above for any $y \in \mathbb {R}^d$ that is bounded away from the points in the sequence $\{x_n\}$, cf. Lemma 4. This, in turn, can be shown if there is a function that takes the value 1 at *y* and zero outside a neighbourhood of *y*, and that belongs to the corresponding native space $\mathcal {N}_{\phi ,m}(\mathbb {R}^d)$ of $\phi$, as defined below, cf. Gutmann [12], Definition 3.10.

#### Definition 1

Let $\mathcal {D}\subset \mathbb {R}^d$, and let $\mathcal {N}_{\phi ,m}(\mathcal {D})$ be the space of functions $f:\mathcal {D}\rightarrow \mathbb {R}$, such that for any $\mathcal {P}_m^d$-unisolvent set $\{x_1,\ldots ,x_n\} \subset \mathcal {D}$ the optimal interpolant $s \in \mathcal {A}_{\phi , m} (\mathcal {D})$ to *f* at these points satisfies

$$\begin{aligned} \Vert s\Vert _\phi \le C_f, \end{aligned}$$

where $C_f$ is a nonnegative constant that only depends on *f*. Then $\mathcal {N}_{\phi ,m}(\mathcal {D})$ is called the native space.

A useful criterion for a function to be in the native space of a radial basis function can be given for surface splines by the following theorem, cf. Gutmann [12], Theorem 3.19.

#### Theorem 4

Let $\phi$ be a conditionally positive definite surface spline of order $m$ from Table 2, and let

$$\begin{aligned} \nu _d = {\left\{ \begin{array}{ll} (d + \nu + 1)/2 &{} \text {if } d+\nu \text { is odd,}\\ (d + \nu )/2 &{} \text {if } d+\nu \text { is even.} \end{array}\right. } \end{aligned}$$

If $f \in C^{\nu _d}(\mathcal {D})$, where (*i*) $\mathcal {D}\subset \mathbb {R}^d$ is compact, or (*ii*) $\mathcal {D}= \mathbb {R}^d$ and *f* has compact support, then $f \in \mathcal {N}_{\phi ,m}(\mathcal {D})$.

Note, however, that the native spaces of multiquadrics, inverse multiquadrics, and Gaussians do not contain any nonzero functions with compact support, as shown by the next corollary, cf. Gutmann [12], Corollary 6.34. It is thus not possible to generalise the convergence proof to these radial basis functions.

#### Corollary 1

Let $\phi$ be a multiquadric, inverse multiquadric, or Gaussian type function of order *m* from Table 2. If $f \in \mathcal {N}_{\phi ,m}(\mathbb {R}^d)$ has compact support, then $f \equiv 0$.

In the case of spline type radial basis functions, Gutmann’s main convergence result, stating that the generated sequence is dense in $\mathcal {X}$, cf. Gutmann [12], Theorem 4.5, can now be formulated in the noisy setup as follows. For a proof of the statement, see Sect. 5.3.

#### Theorem 5

Let $\phi$ be a conditionally positive definite surface spline of order $m$ from Table 2, and let $\{x_n\}$ be the sequence of iterates generated by Algorithm 2. Further, let $s_n^{\gamma _n}$ with $\gamma _n >0$ be the optimal regularised least-squares approximant from $\mathcal {A}_{\phi , m} (\mathcal {X})$ to the data $(x_i,\hat{f}^{(n)} (x_i))$, $i=1,\ldots ,n$, with corresponding weights $w_i$ bounded away from zero. Assume that, for infinitely many $n \in \mathbb {N}$, the choice of $f_n^*$ satisfies (44), where $\tau$, $\varDelta _n$, $\rho$ and $\widetilde{w}_n$ are given as above, and that $n \gamma _n \rightarrow 0$ as $n \rightarrow \infty$. Then, the sequence $\{x_n\}$ is dense in $\mathcal {X}$.

In view of Gutmann [12], Corollary 4.6, we can conclude the following particular convergence result from Theorems 2 and 5, due to the finiteness of the right-hand side in assumption (44) for any $n \in \mathbb {N}$.

#### Corollary 2

Let $\phi$ and $m$ be as in Theorem 5. Further, let *f* be continuous with minimal function value $f^*$, and assume that, for infinitely many $n \in \mathbb {N}$, it holds $f_n^*= -\infty$. Suppose $\epsilon _n \rightarrow 0$ for $n \rightarrow \infty$ in the case of fixed noise and $\max _{1 \le i \le n} \epsilon _i^{(n)} \rightarrow 0$ for $n \rightarrow \infty$ in the case of vanishing iterative noise, and $\gamma _n = {\textbf {o}}(1/n)$ holds. Then, we have $\lim _{n \rightarrow \infty } f ( x^{(n)} ) = f^*$ for the sequence $\{ x^{(n)} \}$ constructed by Algorithm 2.

To derive a further convergence result applying to functions *f* in the native space and under particular assumptions on the error bounds, we first show that for sufficiently large *n* the maximum norm of the approximating surface can be bounded, cf. Gutmann [12], Lemma 4.7, for the equivalent case of interpolation. The lemma below assumes that *f* is from the corresponding native space and uses the norm $\Vert \cdot \Vert _{\mathcal {N}_{\phi ,m}}$ on this native space, as introduced by Schaback [37].

#### Lemma 1

Let $\{x_n\}$ be a sequence in $\mathcal {X}$ with pairwise different points such that $\{x_1,\ldots ,x_{n_0}\}$ is $\mathcal {P}_m^d$-unisolvent. For $n \ge n_0$, let $s_n^{\gamma _n}$ with $\gamma _n > 0$ denote the optimal regularised least-squares approximant to $\hat{f}$ at $x_1,\ldots ,x_n$, where the respective weights $w_1,\ldots ,w_n$ are bounded away from zero. Further, let $f \in \mathcal {N}_{\phi ,m}(\mathcal {X})$, and assume that $n \gamma _n \le n_0 \gamma _{n_0}$ for sufficiently large *n*. Then, for *n* large enough,

47 $$\begin{aligned} \Vert s_n^{\gamma _n}\Vert _{\infty } \le \frac{1}{\sqrt{{\alpha _1}}} \Bigg (\big \Vert f\big \Vert _{\mathcal {N}_{\phi ,m}}^2 + \frac{1}{n \gamma _n} \sum _{i=1}^n w_i \big ( f(x_i) - \hat{f}^{(n)} (x_i) \big )^2\Bigg )^{1/2} + \, \big \Vert f\big \Vert _\infty , \end{aligned}$$

where ${\alpha _1}$ is a constant depending on $x_1,\ldots ,x_{n_0}$.

#### Proof

Fix $n \in \mathbb {N}$, and let *y* be any point in $\mathcal {X}\backslash \{x_1,\ldots ,x_n\}$. For $\gamma _n >0$, let $\tilde{s}_n^{\gamma _n}$ be the optimal regularised least-squares approximant from $\mathcal {A}_{\phi , m} (\mathcal {X})$ to $(x_i,\hat{f}^{(n)} (x_i))$, $i=1,\ldots ,n$, with corresponding weights $w_i$ bounded away from zero, and subject to $\tilde{s}_n^{\gamma _n}(y) = f(y)$. Analogous to the derivation in Sect. 4.2, the approximant can thus be rewritten as

48 $$\begin{aligned} \tilde{s}_n^{\gamma _n}(x) = s_n^{\gamma _n}(x) + \big [ f(y)-s_n^{\gamma _n}(y) \big ] l_n^{\gamma _n}(y,x), \qquad x \in \mathbb {R}^d, \end{aligned}$$

where $l_n^{\gamma _n}(y,{\hspace{1.111pt}\cdot \hspace{1.111pt}})$ is the optimal regularised least-squares approximant to $(x_i,0)$, with respective weights $w_i$, and subject to $l_n^{\gamma _n}(y,y)=1$. Moreover, it follows that

49 $$\begin{aligned}&\gamma _n \Vert \tilde{s}_n^{\gamma _n}\Vert _\phi ^2 + \frac{1}{n}\sum _{i=1}^n w_i \big ( \tilde{s}_n^{\gamma _n}(x_i) - \hat{f}^{(n)} (x_i) \big )^2 \nonumber \\&\quad = \gamma _n \Vert s_n^{\gamma _n}\Vert _\phi ^2 + \frac{1}{n} \sum _{i=1}^n w_i \big ( s_n^{\gamma _n}(x_i) - \hat{f}^{(n)} (x_i)\big )^2 + \big [f(y) - s_n^{\gamma _n}(y) \big ]^2 \gamma _n \mu _n^{\gamma _n}(y), \end{aligned}$$

where the positive function $\mu _n^{\gamma _n}$ of the approximant $l_n^{\gamma _n}(y,{\hspace{1.111pt}\cdot \hspace{1.111pt}})$ is given by (38). Equality (49) thus yields

50 $$\begin{aligned} \big [ f(y) - s_n^{\gamma _n}(y) \big ]^2 \le \frac{\gamma _n \Vert \tilde{s}_n^{\gamma _n}\Vert _\phi ^2 + \frac{1}{n} \sum _{i=1}^n w_i \big (\tilde{s}_n^{\gamma _n}(x_i) - \hat{f}^{(n)} (x_i)\big )^2}{\gamma _n \mu _n^{\gamma _n}(y)}. \end{aligned}$$

The right-hand side of inequality (50) can further be bounded as follows. On the one hand, the optimality of the approximant $\tilde{s}_n^{\gamma _n}$ provides

51 $$\begin{aligned}&\gamma _n \Vert \tilde{s}_n^{\gamma _n}\Vert _\phi ^2 + \frac{1}{n} \sum _{i=1}^n w_i \big ( \tilde{s}_n^{\gamma _n}(x_i) - \hat{f}^{(n)} (x_i) \big )^2 \nonumber \\&\qquad \le \gamma _n \Vert \tilde{s}_n\Vert _\phi ^2 + \frac{1}{n} \sum _{i=1}^n w_i \big ( \tilde{s}_n(x_i) - \hat{f}^{(n)} (x_i) \big )^2 \nonumber \\&\qquad \le \gamma _n \big \Vert f\big \Vert _{\mathcal {N}_{\phi ,m}}^2 + \frac{1}{n } \sum _{i=1}^n w_i \big ( f(x_i) - \hat{f}^{(n)} (x_i) \big )^2, \end{aligned}$$

where $\tilde{s}_n$ is the optimal interpolant to the data $(x_i,f(x_i))$, $i=1,\ldots ,n$, and (*y*, *f*(*y*)), whose semi-norm is bounded by $\Vert f\Vert _{\mathcal {N}_{\phi ,m}}$ as $f \in \mathcal {N}_{\phi ,m}(\mathcal {X})$, see Definition 1. On the other hand, we have for sufficiently large $n \ge n_0$ with $n \gamma _n \le n_0 \gamma _0$ that

$$\begin{aligned} \mu _n^{\gamma _n}(y)&\ge \Vert l_n^{\gamma _{n}}(y,{\hspace{1.111pt}\cdot \hspace{1.111pt}})\Vert _\phi ^2 + \frac{1}{n_0 \gamma _{n_0}} \sum _{i=1}^{n_0} w_i \big (l_n^{\gamma _n}(y,x_i) \big )^2 \\&\ge \Vert l_{n_0}^{\gamma _{n_0}}(y,{\hspace{1.111pt}\cdot \hspace{1.111pt}})\Vert _\phi ^2 + \frac{1}{n_0 \gamma _{n_0}} \sum _{i=1}^{n_0} w_i \big (l_{n_0}^{\gamma _{n_0}}(y,x_i) \big )^2 = \mu _{n_0}^{\gamma _{n_0}}(y), \end{aligned}$$

where $l_{n_0}^{\gamma _{n_0}}(y,{\hspace{1.111pt}\cdot \hspace{1.111pt}})$ with regularisation parameter $\gamma _{n_0} > 0$ is the optimal approximant to $(x_1,0),\ldots ,(x_{n_0},0)$ with respective weights $w_1,\ldots ,w_{n_0}$, and subject to $l_{n_0}^{\gamma _{n_0}}(y,y) = 1$. By Cramer’s rule, the positive function $\mu _{n_0}^{\gamma _{n_0}}$ can then be computed as $\mu _{n_0}^{\gamma _{n_0}}(y) = \det A_{n_0}^{\gamma _{n_0}}/ \det A_{n_0}^{\gamma _{n_0}}(y)$, where the nonsingular matrices $A_{n_0}^{\gamma _{n_0}}$ and $A_{n_0}^{\gamma _{n_0}}(y)$ are given in (39) for $n=n_0$, respectively. Now, $\det A_{n_0}^{\gamma _{n_0}}$ is a nonzero constant and $\det A_{n_0}^{\gamma _{n_0}}(y)$ is bounded on $\mathcal {X}$, as a continuous function. It thus follows that $\mu _{n_0}^{\gamma _{n_0}}(y)$ is bounded away from zero. Hence, there exists a constant ${\alpha _1}> 0$, depending on $x_1,\ldots ,x_{n_0}$ and on $\gamma _{n_0}$, such that

52 $$\begin{aligned} \mu _n^{\gamma _n}(y) \ge {\alpha _1}, \qquad \forall \, y \in \mathcal {X}\backslash \{x_1,\ldots ,x_n\}, \; n \ge n_0. \end{aligned}$$

Consequently, by (51) and (52), we get that inequality (50) reduces to

$$\begin{aligned} \big [ f(y) - s_n^{\gamma _n}(y) \big ]^2 \le \frac{\big \Vert f\big \Vert _{\mathcal {N}_{\phi ,m}}^2 + \frac{1}{n \gamma _n} \sum _{i=1}^n w_i \big ( f(x_i) - \hat{f}^{(n)} (x_i) \big )^2}{{\alpha _1}}, \end{aligned}$$

which, as *f* is bounded on $\mathcal {X}$, results in

53 $$\begin{aligned} \big |s_n^{\gamma _n}(y)\big | \le \frac{1}{\sqrt{{\alpha _1}}} \Bigg (\big \Vert f\big \Vert _{\mathcal {N}_{\phi ,m}}^2 \!+\! \frac{1}{n \gamma _n} \sum _{i=1}^n w_i \big ( f(x_i) \!-\! \hat{f}^{(n)} (x_i) \big )^2\Bigg )^{1/2} \!+\! \big \Vert f\big \Vert _\infty , \end{aligned}$$

for $$y \in \mathcal {X}\backslash \{x_1,\ldots ,x_n\}$$.

Due to the continuous extension of $\mu _n^{\gamma _n}$, inequality (52) also applies at the sample points, cf. Eq. (40). Accordingly, since $\phi$ is assumed to be conditional positive definite and thus continuous, the upper bound in (53) is also valid for $s_n^{\gamma _n}$ at the sample points $x_1,\ldots ,x_n$. $\square$

Under additional assumptions on the scaled weighted sum of squared errors in inequality (47) such that $\{\Vert s_n^{\gamma _n}\Vert _\infty \}$ is bounded uniformly, the following convergence result for sufficiently smooth objective functions *f* can then be established together with Theorems 2 and 5. For the analogous deterministic case, see [12], Corollary 4.8.

#### Corollary 3

Let $\phi$ and $m$ be as in Theorem 5. Further, let $\nu _d$ be as in Theorem 4, $f \in C^{\nu _d}(\mathcal {X})$ with minimal function value $f^*$, and let the sequence $(1/n \gamma _n ) \sum _{i=1}^n w_i (f(x_i) - \hat{f}^{(n)} (x_i))^2\}$ be convergent. Assume that, for infinitely many $n \in \mathbb {N}$, we have

$$\begin{aligned} f_n^*< \min _{y \in \mathcal {X}} \Big [s_n^{\gamma _n}(y) - \tau \big [ \varDelta _n(y) + \widetilde{w}_n^{-1/2}(y) \big ]^{\rho /2} \Big ], \end{aligned}$$

where $\tau$, $\varDelta _n$, $\rho$ and $\widetilde{w}_n$ are given as above, and that $n \gamma _n \rightarrow 0$ as $n \rightarrow \infty$. Then, we have $\lim _{n \rightarrow \infty } f ( x^{(n)} ) = f^*$ for the sequence $\{ x^{(n)} \}$ constructed by Algorithm 2.

#### Remark 6

Note that Lemma 1 may also be formulated for noisy functions $\hat{f}$ in the native space, i.e. for functions with sufficiently well-behaved noise. In this case, $\Vert s_n^{\gamma _n}\Vert _\infty$ can be bounded uniformly by a number that only depends on $x_1,\ldots ,x_{n_0}$, $\gamma _{n_0}$, and $\hat{f}$, such that Corollary 3 holds for $\hat{f} \in \mathcal {N}_{\phi ,m}(\mathcal {X})$.

### Proof of convergence

To prove Theorem 5, we require some lemmas on the behaviour of the functions $\mu _n^{\gamma _n}$, $n \in \mathbb {N}$. The lemmas essentially generalise Lemmas 4.9–4.11 in [12] in order to account for the presence of noise. Correspondingly, the first two lemmas are concerned with the limit of the sequence $\{\mu _n^{\gamma _n}(x_n)\}$.

#### Lemma 2

Let $\phi$ be a conditionally positive definite radial basis function of order $m$ from Table 2, and let $\{z_1,\ldots ,z_k\}$ be a $\mathcal {P}_m^d$-unisolvent set in a compact set $\mathcal {X}\subset \mathbb {R}^d$. Let $\{x_n\}$ and $\{y_n\}$ be convergent sequences in $\mathcal {X}$ that have the same limit $x^*\notin \{z_1,\ldots ,z_k\}$ and satisfy $x_n \ne y_n$, $n \in \mathbb {N}$. Further, let $\tilde{l}_n^{\gamma _n}(x_n,{\hspace{1.111pt}\cdot \hspace{1.111pt}})$ with $\gamma _n > 0$ be the optimal regularised least-squares approximant to the data $(z_1,0),\ldots ,(z_k,0),(y_n,0)$ and subject to $\tilde{l}_n^{\gamma _n}(x_n,x_n)=1$, where the corresponding weights $w_1,\ldots ,w_k,w_n$ are bounded away from zero.

If $n \gamma _n \rightarrow 0$ as $n \rightarrow \infty$, then

54 $$\begin{aligned} \lim _{n \rightarrow \infty } \big [ \Vert y_n - x_n\Vert _2 + w_n^{-1/2} \big ]^\rho \, \tilde{\mu }_n^{\gamma _n}(x_n) = \infty , \end{aligned}$$

where $\tilde{\mu }_n^{\gamma _n}$ is the function defined by (38) for the approximant $\tilde{l}_n^{\gamma _n}(x_n,{\hspace{1.111pt}\cdot \hspace{1.111pt}})$, and where $0 \le \rho < 1$, for $\phi (r)=r$, and $0 \le \rho < 2$, otherwise.

#### Proof

For $\gamma _n > 0$, consider the optimal approximant $\tilde{l}_n^{\gamma _n}(x_n,{\hspace{1.111pt}\cdot \hspace{1.111pt}})$ to $(z_1,0),\ldots ,(z_k,0),(y_n,0)$, with corresponding weights $w_1,\ldots ,w_k,w_n$, and interpolating $(x_n,1)$. For sufficiently large *n*, neither $x_n$ nor $y_n$ is in the set $\{z_1,\ldots ,z_k\}$, so that Cramer’s rule may be applied to compute the function $\tilde{\mu }_n^{\gamma _n}$ associated to $\tilde{l}_n^{\gamma _n}(x_n,{\hspace{1.111pt}\cdot \hspace{1.111pt}})$ by

$$\begin{aligned} \tilde{\mu }_n^{\gamma _n}(x_n) = \frac{\det A_n^{\gamma _n}}{\det A_n^{\gamma _n}(x_n)}, \end{aligned}$$

where the nonsingular matrices $A_n^{\gamma _n}$ and $A_n^{\gamma _n}(x_n)$ are of the form (39) for the points $z_1,\ldots ,z_k,y_k$ and $z_1,\ldots ,z_k,y_k,x_n$, respectively. In particular, the latter matrix can be written as

$$\begin{aligned} A_n^{\gamma _n}(x_n) = \begin{pmatrix} \varPhi + n\gamma _n W^{-1} &{}\quad u_k(y_n) &{}\quad u_k(x_n) &{}\quad P \\ u_k(y_n)^\top &{}\quad \phi (0) + n \gamma _n w_n^{-1} &{}\quad \phi (\Vert y_n - x_n\Vert _2) &{}\quad \pi (y_n)^\top \\ u_k(x_n)^\top &{}\quad \phi (\Vert y_n - x_n\Vert _2) &{}\quad \phi (0) &{}\quad \pi (x_n)^\top \\ P^\top &{}\quad \pi (y_n) &{}\quad \pi (x_n) &{}\quad 0 \end{pmatrix}, \end{aligned}$$

where $\varPhi \in \mathbb {R}^{k \times k}$ and $P \in \mathbb {R}^{k \times {\widetilde{m}}}$ correspond to the interpolation and polynomial basis matrix of $\{z_1,\ldots ,z_k\}$, respectively, $W = {\text {diag}}(w_1,\ldots ,w_k)$, and $u_k(y)= (\phi (\Vert z_1-y\Vert _2),\ldots ,\phi (\Vert z_k-y\Vert _2))^\top$ and $\pi (y) = (p_1(y),\ldots ,p_{{\widetilde{m}}}(y))^\top$ for any $y \in \mathcal {X}$.

By the continuity of the determinant and the assumption $n\gamma _n \rightarrow 0$ as $n \rightarrow \infty$ with weights bounded away from zero, it follows that $\lim _{n \rightarrow \infty } \det A_n^{\gamma _n} = \det A^{*} \ne 0$, where $A^{*}$ denotes the nonsingular interpolation matrix given in form of the left-hand side of (8) for the points $z_1,\ldots ,z_k,x^{*}$. In order to show assertion (54), it therefore remains to consider expression

55 $$\begin{aligned} \big [\Vert y_n - x_n\Vert _2 + w_n^{-1/2} \big ]^{-\rho } \, \det A_n^{\gamma _n}(x_n), \end{aligned}$$

for which we show in the following that it converges to zero as $n \rightarrow \infty$. First note that the $(k+1)$-th and $(k+2)$-th rows of the matrix $A_n^{\gamma _n}(x_n)$, given by

$$\begin{aligned}&\begin{array}{lllll} \big ( u_k(y_n)^{\top } &{} \phi (0) + n \gamma _n w_n^{-1} &{} \phi (\Vert y_n - x_n\Vert _2) &{} \pi (y_n)^\top \big ), &{} \text { and } \\ \big ( u_k(x_n)^\top &{} \phi (\Vert y_n - x_n\Vert _2) &{} \phi (0) &{} \pi (x_n)^\top \big ), \end{array} \end{aligned}$$

have the same limit for $n \rightarrow \infty$, as the weights are bounded away from zero and $n \gamma _n \rightarrow 0$ for $n \rightarrow \infty$. Consequently, $\det A_n^{\gamma _n}(x_n) \rightarrow 0$ as $n \rightarrow \infty$, and hence, for $\rho =0$, assertion (54) follows immediately.

For $\rho > 0$, note that the determinant of $A_n^{\gamma _n}(x_n)$ does not change if the $(k+1)$-th row of the matrix $A_n^{\gamma _n}(x_n)$ is replaced by the difference between the $(k+1)$-th and the $(k+2)$-th row, and, subsequently, the $(k+1)$-th column is replaced by the difference between the $(k+1)$-th and the $(k+2)$-th column. Therefore, $\det A_n^{\gamma _n}(x_n)$ can equally be computed asTo deduce the convergence of expression (55) to zero, we then divide the $(k+1)$-th row and the $(k+1)$-th column of the latter determinant by $[\Vert y_n - x_n\Vert _2 + w_n^{-1/2}]^{\rho /2}$, and make the following remarks on the newly formed $(k+1)$-th column.

For all choices of $\phi$, the functions $\phi (\Vert z_i - {\hspace{1.111pt}\cdot \hspace{1.111pt}}\Vert _2)$, $i=1,\ldots ,k$, are Lipschitz continuous on $\mathcal {X}$. This implies for $\rho < 2$ that

$$\begin{aligned} \lim _{n \rightarrow \infty } \frac{\phi (\Vert z_i-y_n\Vert _2)-\phi (\Vert z_i-x_n\Vert _2)}{\big [\Vert y_n - x_n\Vert _2 + w_n^{-1/2}\big ]^{\rho /2}} = 0, \qquad i=1,\ldots ,k, \end{aligned}$$

such that $u_k(y_n) - u_k(x_n) \rightarrow 0$ as $n \rightarrow \infty$. Similarly, for the same choice of $\rho$, the Lipschitz continuity of the polynomials yields

$$\begin{aligned} \lim _{n \rightarrow \infty } \frac{p_j(y_n) - p_j(x_n)}{\big [\Vert y_n - x_n\Vert _2 + w_n^{-1/2}\big ]^{\rho /2}} = 0, \qquad j=1,\ldots ,{\widetilde{m}}, \end{aligned}$$

resulting in $\pi (y_n) - \pi (x_n) \rightarrow 0$ as $n \rightarrow \infty$. Further, we have

$$\begin{aligned} \lim _{n \rightarrow \infty } \frac{\phi (\Vert y_n - x_n\Vert _2) - \phi (0)}{\big [\Vert y_n - x_n\Vert _2 + w_n^{-1/2}\big ]^\rho } = 0, \end{aligned}$$

for $\rho < \nu$ in the case of surface splines and for $\rho < 2$ in the other cases. This follows directly in the case of surface splines, due to their form, and by the second order Taylor expansion in the other cases, as $\phi ^\prime (0) = 0$ and $\phi ^{\prime \prime }(r)$ is bounded for small *r*.

Eventually, by assuming that $n\gamma _n \rightarrow 0$ as $n \rightarrow \infty$ and since $w_n$ is bounded away from zero, we observe for $\rho < 2$,

$$\begin{aligned} \lim _{n \rightarrow \infty } \frac{n \gamma _n w_n^{-1}}{\big [\Vert y_n - x_n\Vert _2 + w_n^{-1/2}\big ]^\rho } = 0. \end{aligned}$$

Altogether, we therefore have that expression (55) converges for the given choices of $\rho$ to zero as $n \rightarrow \infty$, proving that assertion (54) also holds in case $\rho >0$. $\square$

#### Lemma 3

Let $\phi$ and $m$ be as in Lemma 2, where $\rho$ takes a value as indicated. Let $\{x_n\}$ be a sequence in $\mathcal {X}$ with pairwise different points such that $\{x_1,\ldots ,x_{n_0}\}$ is $\mathcal {P}_m^d$-unisolvent. For any $y \in \mathcal {X}\backslash \{x_1,\ldots ,x_n\}$, let $l_n^{\gamma _n}(y,{\hspace{1.111pt}\cdot \hspace{1.111pt}})$ with $\gamma _n>0$ be the optimal regularised least-squares approximant to the data $(x_1,0),\ldots ,(x_n,0)$ and subject to $l_n^{\gamma _n}(y,y)=1$, where the corresponding weights $w_1,\ldots ,w_n$ are bounded away from zero. If $n \gamma _n \rightarrow 0$ as $n \rightarrow \infty$, then for every convergent subsequence $\{x_{n_k}\}_{k \in \mathbb {N}}$ of $\{x_n\}$ it holds

$$\begin{aligned} \lim _{k \rightarrow \infty } \big [ \varDelta _{n_k-1}(x_{n_k}) + \widetilde{w}_{n_k-1}^{-1/2}(x_{n_k}) \big ]^\rho \, \mu _{n_k-1}^{\gamma _{n_k-1}}(x_{n_k}) = \infty , \end{aligned}$$

where $\mu _{n_k-1}^{\gamma _{n_k-1}}$, $\varDelta _{n_k-1}$, and $\widetilde{w}_{n_k-1}$ are the functions given by (38), (45), and (46), respectively, for $n=n_k-1$.

#### Proof

For $n \ge 2$, let $i(x_n) = {\hbox {arg min}}_{1 \le i \le n-1} \Vert x_n - x_i\Vert _2$, where we choose the largest $i(x_n)$ among the minimising indices if ${\hbox {arg min}}$ is not unique, and let the sequence $\{y_n\}_{n \in \mathbb {N}}$ be defined as

$$\begin{aligned} y_n := {\left\{ \begin{array}{ll} x_2, &{} n=1, \\ x_{i(x_n)}, &{} n \ge 2. \end{array}\right. } \end{aligned}$$

Further, let $\{x_{n_k}\}$ be any subsequence of $\{x_n\}$ that converges to a point $x^*\in \mathcal {X}$. The choice of $\{y_n\}$ and convergence thus yield $\lim _{k \rightarrow \infty } \Vert x_{n_k} - y_{n_k}\Vert _2 = 0$. Also note that there always exists a $\mathcal {P}_m^d$-unisolvent set $\{\bar{x}_1,\ldots ,\bar{x}_l\}$, $l \in \mathbb {N}$, in the sequence $\{x_n\}$ that does not contain the limit point $x^*$. If $x^*= x_i$ for some $i \in \{1,\ldots ,n_0\}$, then we can pick $x_{n_i}$ in a neighbourhood of $x^*$ such that the initial set $\{x_1,\ldots ,x_{i-1},x_{n_i},x_{i+1},\ldots ,x_{n_0}\}$ is $\mathcal {P}_m^d$-unisolvent.

For sufficiently large $k \in \mathbb {N}$ such that $y_{n_k} \notin \{\bar{x}_1,\ldots ,\bar{x}_l\}$ and for any $y \in \mathcal {X}\backslash \{x_1,\ldots ,x_{n_k-1}\}$, let $\bar{l}_k^{\gamma _k}(y,{\hspace{1.111pt}\cdot \hspace{1.111pt}})$ with $\gamma _k > 0$ be the optimal regularised least-squares approximant to the data $(\bar{x}_1,0),\ldots ,(\bar{x}_l,0)$, $(y_{n_k},0)$, with corresponding weights $\hspace{0.83328pt}\overline{\hspace{-0.83328pt}w\hspace{-0.83328pt}}\hspace{0.83328pt}_1,\ldots ,\hspace{0.83328pt}\overline{\hspace{-0.83328pt}w\hspace{-0.83328pt}}\hspace{0.83328pt}_l$, $w_{n_k}$ bounded away from zero, and subject to $\bar{l}_k^{\gamma _k}(y,y) = 1$. Likewise, let $l_{n_k-1}^{\gamma _{n_k-1}}(y,{\hspace{1.111pt}\cdot \hspace{1.111pt}})$ with $\gamma _{n_k-1} > 0$ be the optimal regularised least-squares approximant to $(x_1,0),\ldots ,(x_{n_k-1},0)$, with corresponding weights $w_1,\ldots ,w_{n_k-1}$ bounded away from zero, and subject to $l_{n_k-1}^{\gamma _{n_k-1}}(y,y)=1$. Observe that, for *k* large enough, $l_{n_k-1}^{\gamma _{n_k-1}}(y,{\hspace{1.111pt}\cdot \hspace{1.111pt}})$ approximates $(\bar{x}_i,0)$, $i=1,\ldots ,l$, and $(y_{n_k},0)$, along with their given weights and subject to the same interpolation condition. Hence, for sufficiently large *k*, the functions $\bar{\mu }_k^{\gamma _k}$ and $\mu _{n_k-1}^{\gamma _{n_k-1}}$ associated to $\bar{l}_k^{\gamma _k}(y,{\hspace{1.111pt}\cdot \hspace{1.111pt}})$ and $l_{n_k-1}^{\gamma _{n_k-1}}(y,{\hspace{1.111pt}\cdot \hspace{1.111pt}})$ via (38), respectively, and the optimality of $\bar{l}_k^{\gamma _k}(y,{\hspace{1.111pt}\cdot \hspace{1.111pt}})$ imply

56 $$\begin{aligned} \begin{aligned} \bar{\mu }_k^{\gamma _k}(y)&=\big \Vert \bar{l}_k^{\gamma _k}(y,{\hspace{1.111pt}\cdot \hspace{1.111pt}})\big \Vert _\phi ^2 + \frac{1}{(l+1) \gamma _k} \Bigg [ \sum _{i=1}^l \hspace{0.83328pt}\overline{\hspace{-0.83328pt}w\hspace{-0.83328pt}}\hspace{0.83328pt}_i \big ( \bar{l}_k^{\gamma _k}(y,\bar{x}_i) \big )^2 + w_{n_k} \big ( \bar{l}_k^{\gamma _k}(y,y_{n_k}) \big )^2 \Bigg ] \\&\le \big \Vert l_{n_k-1}^{\gamma _{n_k-1}}(y,{\hspace{1.111pt}\cdot \hspace{1.111pt}})\big \Vert _\phi ^2 + \frac{1}{(l+1) \gamma _k} \, \sum _{i=1}^{n_k-1} w_i \big (l_{n_k-1}^{\gamma _{n_k-1}}(y,x_i) \big )^2 \\&\le \mu _{n_k-1}^{\gamma _{n_k-1}}(y), \end{aligned} \end{aligned}$$

where the last inequality follows from the assumption that $n \gamma _n \rightarrow 0$ as $n \rightarrow \infty$.

Eventually, by definition of the sequence $\{y_n\}$ and applying Lemma 2 with the set of points $\{z_1,\ldots ,z_k\}$ being $\{\bar{x}_1,\ldots ,\bar{x}_l\}$, the weights $w_1\ldots ,w_k$ being replaced by $\hspace{0.83328pt}\overline{\hspace{-0.83328pt}w\hspace{-0.83328pt}}\hspace{0.83328pt}_1,\ldots ,\hspace{0.83328pt}\overline{\hspace{-0.83328pt}w\hspace{-0.83328pt}}\hspace{0.83328pt}_l$, and setting $n = n_k$, we obtain

$$\begin{aligned}&\lim _{k \rightarrow \infty } \big [ \varDelta _{n_k-1}(x_{n_k}) + \widetilde{w}_{n_k-1}^{-1/2}(x_{n_k}) \big ]^\rho \, \bar{\mu }_k^{\gamma _k}(x_{n_k}) \\&\qquad = \lim _{k \rightarrow \infty } \big [ \Vert x_{n_k} - y_{n_k}\Vert _2 + w_{n_k}^{-1/2} \big ]^\rho \, \bar{\mu }_k^{\gamma _k}(x_{n_k}) = \infty , \end{aligned}$$

for the given choice of $\rho$. Consequently, by setting $y=x_{n_k}$ in (56), it follows that $[\varDelta _{n_k-1}(x_{n_k}) + \widetilde{w}_{n_k-1}^{-1/2}(x_{n_k})]^\rho \, \mu _{n_k-1}^{\gamma _{n_k-1}}(x_{n_k})$ tends to infinity for $k \rightarrow \infty$, as claimed. $\square$

Akin to [12], Lemma 4.11, the next lemma states that the sequence $\{\mu _n^{\gamma _n}(y)\}$ is uniformly bounded if *y* is bounded away from the points in the sequence $\{x_n\}$. Note that this result only holds for surface splines, as Theorem 4 is required.

#### Lemma 4

Let $\phi$ be a conditionally positive definite surface spline of order $m$ from Table 2, and let $\{x_n\}$ be a sequence in $\mathbb {R}^d$ with pairwise different points such that $\{x_1,\ldots ,x_{n_0}\}$ is $\mathcal {P}_m^d$-unisolvent. Further, let $y_0 \in \mathbb {R}^d$ satisfy $\Vert y_0 - x_n\Vert _2 \ge \delta$, $n \in \mathbb {N}$, for some $\delta >0$. Then, there exists $C> 0$, depending only on $y_0$ and $\delta$, such that

$$\begin{aligned} \mu _n^{\gamma _n}(y_0) \le C, \qquad \forall \, n \ge n_0, \end{aligned}$$

where $\mu _n^{\gamma _n}$ with $\gamma _n > 0$ is the function given by (38).

#### Proof

Let $B_\delta (y_0) = \{x \in \mathbb {R}^d: \Vert x-y_0\Vert _2 < \delta \}$. There exists a compactly supported function $\varphi \in C^\infty (\mathbb {R}^d)$ that takes the value 1 at $y_0$ and 0 on $\mathbb {R}^d \backslash B_\delta (y_0)$. It follows from Theorem 4 that $\varphi \in \mathcal {N}_{\phi ,m}(\mathbb {R}^d)$.

For any $n \ge n_0$, let $l_n(y_0,{\hspace{1.111pt}\cdot \hspace{1.111pt}})$ be the optimal interpolant to the data $(x_1,0),\ldots ,(x_n,0)$ and $(y_0,1)$, such that $l_n(y_0,x_i) = \varphi (x_i) = 0$, $i=1,\ldots ,n$, and $l_n(y_0,y_0) = \varphi (y_0) = 1$. Similarly, for any $n \ge n_0$, let $l_n^{\gamma _n}(y_0,{\hspace{1.111pt}\cdot \hspace{1.111pt}})$ with $\gamma _n >0$ denote the optimal regularised least-squares approximant to $(x_1,0),\ldots ,(x_n,0)$, with corresponding weights $w_1,\ldots ,w_n$, and subject to $l_n^{\gamma _n}(y_0,y_0)=1$. By definition of $\mu _n^{\gamma _n}$ and the optimality of $l_n^{\gamma _n}(y_0,{\hspace{1.111pt}\cdot \hspace{1.111pt}})$, we then have

$$\begin{aligned} \mu _n^{\gamma _n}(y_0)&\le \Vert l_n(y_0,{\hspace{1.111pt}\cdot \hspace{1.111pt}})\Vert _\phi ^2 + \frac{1}{n\gamma _n}\sum _{i=1}^n w_i \big ( l_n(y_0,x_i) \big )^2 \\&= \Vert l_n(y_0,{\hspace{1.111pt}\cdot \hspace{1.111pt}})\Vert _\phi ^2, \end{aligned}$$

which is bounded by $C:= \Vert \varphi \Vert _{\mathcal {N}_{\phi ,m}}^2$, see Definition 1. $\square$

By using the lemmas above, we can now provide the main proof of Theorem 5, stating that the sequence generated by Algorithm 2 is dense in $\mathcal {X}$. Because of the established similarity of the algorithm to Gutmann’s RBF method, the proof follows the main lines of the proof of Theorem 4.5 in [12].

#### Proof of Theorem 5

Assume that there is $y_0 \in \mathcal {X}$ and $\delta >0$, such that $B_\delta (y_0) = \{ x \in \mathbb {R}^d: \Vert x-y_0\Vert _2 < \delta \}$ does not contain any $x_n$, $n \in \mathbb {N}$. According to the iteration step of Algorithm 2, it then holds

$$\begin{aligned} g_n^{\gamma _n}(x_{n+1}) \le g_n^{\gamma _n}(y_0), \qquad n \ge n_0, \end{aligned}$$

where $\gamma _n > 0$. Moreover, since $f_n^*$ is assumed to satisfy condition (44) for infinitely many $n \in \mathbb {N}$, there exists a subsequence $\{x_{n_k}\}_{k \in \mathbb {N}}$ such that

$$\begin{aligned} s_{n_k-1}^{\gamma _{n_k-1}}(x_{n_k}) - f_{n_k-1}^*> \tau \big \Vert s_{n_k-1}^{\gamma _{n_k-1}}\big \Vert _\infty \big [ \varDelta _{n_k-1}(x_{n_k}) + \widetilde{w}_{n_k-1}^{-1/2}(x_{n_k}) \big ]^{\rho /2}, \end{aligned}$$

for the specified quantities $\tau$ and $\rho$, and the functions $\varDelta _{n_k-1}$ and $\widetilde{w}_{n_k-1}$ as given by (45) and (46), respectively, for $n=n_k-1$. Now, the sequence $\{x_{n_k}\}$ has a convergent subsequence which, without loss of generality, shall be denoted again by $\{x_{n_k}\}$. Since each $x_{n_k}$, $k \in \mathbb {N}$, minimises $g_{n_k-1}$ on $\mathcal {X}\backslash \{x_1,\ldots ,x_{n_k-1}\}$, the same reasoning as in the proof of Theorem 7 in [10], inequality (A.11) to (A.12), then leads to the inequality

57 $$\begin{aligned}&\mu _{n_k-1}^{\gamma _{n_k-1}}(x_{n_k}) \big [ \varDelta _{n_k-1}(x_{n_k}) + \widetilde{w}_{n_k-1}^{-1/2}(x_{n_k}) \big ]^\rho \nonumber \\&\qquad \le \mu _{n_k-1}^{\gamma _{n_k-1}}(y_0) \bigg [ \big [ \varDelta _{n_k-1}(x_{n_k}) + \widetilde{w}_{n_k-1}^{-1/2}(x_{n_k}) \big ]^{\rho /2} + \frac{2}{\tau } \bigg ]^2, \end{aligned}$$

which renders a contradiction by virtue of Lemmas 2–4. In particular, on the one hand, Lemma 3 reveals that the left-hand side of (57) converges to infinity for $k \rightarrow \infty$. On the other hand, Lemma 4 shows that $\mu _n^{\gamma _n}(y_0)$ is bounded above by some constant independent of *n*, which together with the uniform boundedness of $\varDelta _{n_k-1}(x_{n_k})$ on $\mathcal {X}$ and the weights being bounded away from zero implies that the right-hand side of inequality (57) is bounded above by a constant independent of *k*. Hence, due to this contradiction, we can deduce that $B_\delta (y_0)$ must contain a point of the sequence $\{x_n\}$, so that, eventually, $\{x_n\}$ is dense in the compact set $\mathcal {X}$. $\square$

## Some illustrative numerical results

In this section, we provide some illustrative numerical results by employing the RBF method for noisy objective functions on a simple test problem. Specifically, we consider the underlying deterministic objective function

$$\begin{aligned} f(x) = -(1.4 - 3x) \sin (18x), \quad x \in [0,1.1]. \end{aligned}$$

**Fig. 1:**
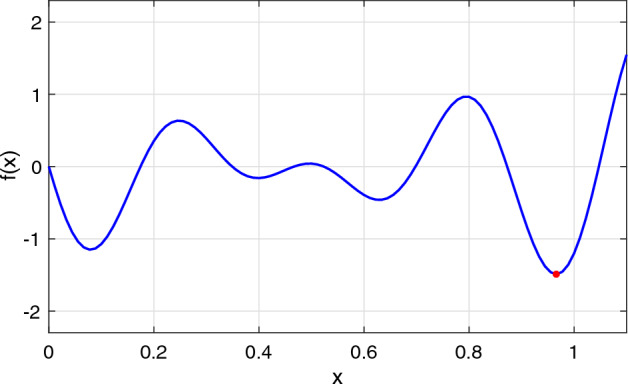
Objective function *f* and global minimum (red point) (colour figure online)

Note that *f* is continuous and nonconvex with a global minimum of $f^*\approx -1.489072$, attained at $x^*\approx 0.966086$, see Fig. 1.

We consider the following two noise models:

1. Fixed noise: we assume that the *i*-th function evaluation of *f*, $i=1,\ldots ,n$, is perturbed such that
  $$\begin{aligned} \hat{f}(x_i) = f(x_i) + \bar{\epsilon }(x_i), \qquad \bar{\epsilon }(x_i) \sim \mathcal {U}\big ([-\epsilon _i, \epsilon _i]\big ), \end{aligned}$$
  where the error bound $\epsilon _i$ is given by $\epsilon _i = 0.5 \cdot i^{-0.4}$.
2. Vanishing iterative noise: we assume that in iteration *n*, the *i*-th function evaluation of *f*, $i=1,\ldots ,n$, is perturbed by
  $$\begin{aligned} \hat{f}^{(n)}(x_i) = f(x_i) + \bar{\epsilon }(x_i), \qquad \bar{\epsilon }(x_i) \sim \mathcal {U}\big ([-\epsilon ^{(n)}, \epsilon ^{(n)}]\big ), \end{aligned}$$
  where the error bound $\epsilon ^{(n)}$ is given by $\epsilon ^{(n)} = 0.5 \cdot n^{-0.4}$. In particular, we thus use in each iteration *n* the same error bounds for all function evaluations, i.e. we have $\epsilon _i^{(n)} = \epsilon ^{(n)}$ for $i=1,\ldots ,n$.

**Fig. 2:**
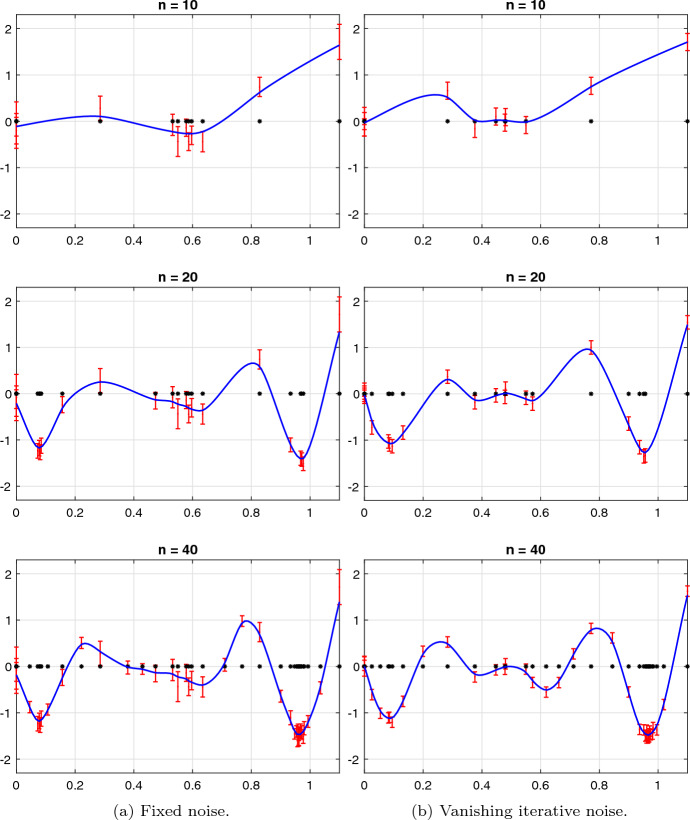
The sample points $x_i$, their observed function values $\hat{f}(x_i)$ and $\hat{f}^{(n)}(x_i)$ with associated error bounds $\epsilon _i$ and $\epsilon ^{(n)}$, respectively, as well as the response surfaces $s_n^{\gamma _n}$ of the RBF method for noisy objective functions after $n=10$, $n=20$, and $n=40$ iterations

To minimise the noisy objective functions, we choose the thin plate spline radial basis function $\phi (r) = r^2 \log {r}$ and initialise the RBF method for noisy objective functions at the end points and the midpoint of the considered interval. We set the regularisation parameter $\gamma _n$ in both noise models by approximately solving the auxiliary problem (31) via a backtracking strategy and set the target value to $f_n^*= \min _{y \in \mathcal {X}} s_n^{\gamma _n}(y) - w_c (\max _{1\le i\le n}{\hat{f}^{(n)}(x_i)} - \min _{1\le i\le n}{\hat{f}^{(n)}(x_i)})$, where $w_c$ cycles through the sequence (1, 0.56, 0.25, 0.06, 0) to balance between global and local search. The subproblems of minimising $s_n^{\gamma _n}$ and $-\log {h_n^{\gamma _n}}$ are solved by the DIRECT algorithm [21].

Results obtained are illustrated in Fig. 2 in form of the points sampled, their corresponding observed noisy function values with prespecified error bounds, as well as the resulting response surfaces for the fixed and vanishing iterative noise models, respectively. In both cases, we can observe that the method is able to recover the global behaviour of the objective function *f* by means of the response surface reasonably well after a certain number of iterations. In particular, we can see by the sampled points that the method successfully balances between global search (by select new points in unexplored regions of the domain) and local search to approximate the global minimum.

## Conclusions

In this paper, we have addressed the global optimisation of an expensive and noisy objective function where observed function values are assumed to lie within error bounds. Based on Gutmann’s original RBF method for minimising a deterministic objective function, relying on radial basis function interpolation, we have first discussed common approaches of radial basis function approximations for integration into a response surface method. Arguing in favour of regularised least-squares approximants, we then have presented a noisy RBF method that constructs the smoothest possible response surfaces that stay within the given error bounds at the evaluated points, and determines new evaluation points by minimising a regularised least-squares criterion in terms of a target value. Further on, we have established convergence of the noisy RBF method to the global minimum of any continuous function, under some additional assumptions on the error bounds, and provided relevant convergence results. Finally, we have provided a numerical illustration of our RBF method for noisy objective functions by considering a simple test problem. Future work will include the assessment of the proposed method on various academic and real-world test functions.

## Appendix

This section summarises the convergence results of the RBF method for deterministic objective functions as detailed in Gutmann [12], Section 4.2 (and Gutmann [10], Section 4).^2^ These results serve as main basis for establishing convergence of the RBF method for noisy objective functions in Sect. 5 of this paper.

In order to show convergence of a global optimisation algorithm to the global minimum of any continuous function on a compact set, it is required that the generated sequence of sample points is dense, see Törn and Žilinskas [43], Theorem 1.3. Applied to Gutmann’s RBF method, this can thus be stated as follows, see Gutmann [12], Theorem 4.4 (or Gutmann [10], Theorem 4).

### Theorem 6

Algorithm 1 converges for every continuous function *f* if and only if it generates a sequence of points that is dense in $\mathcal {X}$.

### Main convergence result

The convergence of the RBF method does not allow a free choice of radial basis function. This is because the proof relies on the existence of a compactly supported, infinitely differentiable function that takes the value 1 at a point $y \in \mathbb {R}^d$ and 0 outside a neighbourhood of *y*, and that belongs to the corresponding native space of the chosen radial basis function (see Definition 1). For spline type radial basis functions, cf. Table 2, it can be shown that their native space contains sufficiently smooth and compactly supported functions, see Theorem 4. However, the native spaces of multiquadric, inverse multiquadric, and Gaussian radial basis functions do not contain any nonzero functions with compact support, cf. Corollary 1. Consequently, Gutmann’s convergence proof cannot be extended to these kind of radial basis functions.

Moreover, the convergence of the method is established under the assumption that the target values $f_n^*$ are set sufficiently low compared to the interpolating surfaces $s_n$. More specifically, it is required that, for infinitely many $n \in \mathbb {N}$, the target values $f_n^*$ satisfy

58 $$\begin{aligned} f_n^*< \min _{y \in \mathcal {X}} \Big [ s_n(y) - \tau \Vert s_n\Vert _\infty \varDelta _n^{\rho /2}(y) \Big ], \end{aligned}$$

where $\tau >0$ and $\rho \ge 0$ are constants with $\rho < 1$, for $\phi (r) = r$, and $\rho < 2$, otherwise, and $\varDelta _n$ denotes the minimum distance function defined in (45).

Gutmann’s main convergence result, stating that the generated sequence is dense in $\mathcal {X}$, can then be formulated for surface splines as follows, see Gutmann [12], Theorem 4.5 (or Gutmann [10], Theorem 7).

#### Theorem 7

Let $\phi$ be a conditionally positive definite surface spline of order $m$ from Table 2, and let $\{x_n\}$ be the sequence of iterates generated by Algorithm 1. Further, let $s_n$ be the optimal interpolant from $\mathcal {A}_{\phi , m} (\mathcal {X})$ to the data $(x_i,f (x_i))$, $i=1,\ldots ,n$. Assume that, for infinitely many $n \in \mathbb {N}$, the choice of $f_n^*$ satisfies (58), where $\tau$, $\varDelta _n$, and $\rho$ are given as above. Then, the sequence $\{x_n\}$ is dense in $\mathcal {X}$.

The proof of Theorem 7 is based on the following lemmas, cf. Gutmann [12], Lemmas 4.9–4.11 (or [10], Lemmas 12–14). Essentially, the first two lemmas are concerned with the limit of the sequence $\{\mu _n(x_n)\}$, whereas the third lemma states that the sequence $\{\mu _n(y)\}$ is uniformly bounded if *y* is bounded away from the points in the sequence $\{x_n\}$. Note that the latter result only holds for surface splines, as Theorem 4 is required.

#### Lemma 5

Let $\phi$ be a conditionally positive definite radial basis function of order $m$ from Table 2, and let $\{z_1,\ldots ,z_k\}$ be a $\mathcal {P}_m^d$-unisolvent set in a compact set $\mathcal {X}\subset \mathbb {R}^d$. Let $\{x_n\}$ and $\{y_n\}$ be convergent sequences in $\mathcal {X}$ that have the same limit $x^*\notin \{z_1,\ldots ,z_k\}$ and satisfy $x_n \ne y_n$, $n \in \mathbb {N}$. Further, let $\tilde{l}_n(x_n,{\hspace{1.111pt}\cdot \hspace{1.111pt}})$ be the optimal interpolant to the data $(z_1,0),\ldots ,(z_k,0),(y_n,0)$, and $(x_n,1)$. Then

$$\begin{aligned} \lim _{n \rightarrow \infty } \Vert y_n - x_n\Vert _2^\rho \, \tilde{\mu }_n(x_n) = \infty , \end{aligned}$$

where $\tilde{\mu }_n$ is the function defined by (15) for the interpolant $\tilde{l}_n(x_n,{\hspace{1.111pt}\cdot \hspace{1.111pt}})$, and where $0 \le \rho < 1$, for $\phi (r)=r$, and $0 \le \rho < 2$, otherwise.

#### Lemma 6

Let $\phi$ and $m$ be as in Lemma 5, where $\rho$ takes a value as indicated. Let $\{x_n\}$ be a sequence in $\mathcal {X}$ with pairwise different points such that $\{x_1,\ldots ,x_{n_0}\}$ is $\mathcal {P}_m^d$-unisolvent. For any $y \in \mathcal {X}\backslash \{x_1,\ldots ,x_n\}$, let $l_n(y,{\hspace{1.111pt}\cdot \hspace{1.111pt}})$ be the optimal interpolant to the data $(x_1,0),\ldots ,(x_n,0)$, and (*y*, 1). Then, for every convergent subsequence $\{x_{n_k}\}_{k \in \mathbb {N}}$ of $\{x_n\}$, it holds

$$\begin{aligned} \lim _{k \rightarrow \infty } \varDelta _{n_k-1}^\rho (x_{n_k}) \, \mu _{n_k-1}(x_{n_k}) = \infty , \end{aligned}$$

where $\mu _{n_k-1}$ and $\varDelta _{n_k-1}$ are the functions given by (15) and (45), respectively, for $n=n_k-1$.

#### Lemma 7

Let $\phi$ be a conditionally positive definite surface spline of order $m$ from Table 2, and let $\{x_n\}$ be a sequence in $\mathbb {R}^d$ with pairwise different points such that $\{x_1,\ldots ,x_{n_0}\}$ is $\mathcal {P}_m^d$-unisolvent. Further, let $y_0 \in \mathbb {R}^d$ satisfy $\Vert y_0 - x_n\Vert _2 \ge \delta$, $n \in \mathbb {N}$, for some $\delta >0$. Then, there exists $C> 0$, depending only on $y_0$ and $\delta$, such that

$$\begin{aligned} \mu _n(y_0) \le C, \qquad \forall \, n \ge n_0, \end{aligned}$$

where $\mu _n$ is the function given by (15).

### Specific convergence results

From Theorems 6 and 7, a particular convergence result can be established by observing that the right-hand side in assumption (58) is finite for any $n \in \mathbb {N}$, see Gutmann [12], Corollary 4.6 (or Gutmann [10], Corollary 8).

#### Corollary 4

Let $\phi$ and $m$ be as in Theorem 5. Further, let *f* be continuous and assume that, for infinitely many $n \in \mathbb {N}$, it holds $f_n^*= -\infty$. Then, Algorithm 1 converges.

A further convergence result applies to functions *f* that lie in the native space $\mathcal {N}_{\phi ,m}(\mathcal {X})$ of a chosen radial basis function $\phi$, as in this case it can be shown that $\{\Vert s_n\Vert _\infty \}$ is uniformly bounded, see Gutmann [12], Lemma 4.7 (or Gutmann [10], Lemma 9).

#### Lemma 8

Let *f* be in $\mathcal {N}_{\phi ,m}(\mathcal {X})$. Further, let $\{x_n\}$ be a sequence in $\mathcal {X}$ with pairwise different points such that $\{x_1,\ldots ,x_{n_0}\}$ is $\mathcal {P}_m^d$-unisolvent. For $n \ge n_0$, let $s_n$ denote the optimal interpolant to *f* at $x_1,\ldots ,x_n$. Then,

$$\begin{aligned} \Vert s_n\Vert _{\infty } \le \frac{1}{\sqrt{{\alpha _1}}} \big \Vert f\big \Vert _{\mathcal {N}_{\phi ,m}} + \, \big \Vert f\big \Vert _\infty , \end{aligned}$$

where ${\alpha _1}$ is a constant depending on $x_1,\ldots ,x_{n_0}$.

By Lemma 8, the following convergence result can then be obtained from Theorems 6 and 7 for sufficiently smooth objective functions *f*, see Gutmann [12], Corollary 4.8 (or Gutmann [10], Corollary 11).

#### Corollary 5

Let $\phi$ and $m$ be as in Theorem 7. Further, let $\nu _d$ be as in Theorem 4 and $f \in C^{\nu _d}(\mathcal {X})$. Assume that, for infinitely many $n \in \mathbb {N}$, we have

$$\begin{aligned} f_n^*< \min _{y \in \mathcal {X}} \Big [s_n(y) - \tau \varDelta _n^{\rho /2}(y) \Big ], \end{aligned}$$

where $\tau$, $\varDelta _n$, and $\rho$ are given as above. Then, Algorithm 1 converges.

## Footnotes

1 Note that the coefficients $\alpha _i (y), \beta (y), b_j (y)$ also depend on the parameter $\gamma _n$. To keep the notation simple, we will denote the dependency on $\gamma _n$ only for the approximant $s_n^{\gamma _n}$ and for the function $l_n^{\gamma _n}(y,{\hspace{1.111pt}\cdot \hspace{1.111pt}})$.
